# A Fast $B_{1}^{+}$‐Driven RF Co‐Simulation Optimization Method for RF Coil Arrays

**DOI:** 10.1002/mrm.70603

**Published:** 2026-09-29

**Authors:** Mazin M. Mustafa, Ethan Polcyn, Matt Waks, Alexander Bratch, Gregory J. Metzger, Xiufeng Li, Yigitcan Eryaman

**Affiliations:** ^1^ Center for Magnetic Resonance Research University of Minnesota at Twin Cities Minneapolis Minnesota USA

**Keywords:** Co‐simulation, MRI, optimization, RF coil, RF safety

## Abstract

**Purpose:**

To generate accurate EM simulation models of RF coil arrays, improve coil safety validation procedures, and improve RF power management without sacrificing patient safety.

**Methods:**

A fast, customized RF co‐simulation formulation was developed for pTx RF coils, enabling the use of experimentally measured $B_{1}^{+}$ maps to optimize the values of lumped elements, phase shifters, and attenuators in the EM simulation model, and demonstrate in a phantom. The proposed method was tested on an 8‐channel pTx knee coil at 7T and a 16‐channel pTx head coil at 10.5T. An RF co‐simulation optimization method that relies solely on matching the simulated and experimental S‐matrices was also implemented as a baseline. 50,000 random RF shims were used to evaluate the $B_{1}^{+}$ error for both co‐simulation optimization methods.

**Results:**

The proposed $B_{1}^{+}$‐driven co‐simulation optimization method generated accurate $B_{1}^{+}$ maps when compared to experimentally measured $B_{1}^{+}$ maps. The proposed method reduced the average NRMSE of the EM modeling against the measurements from 48.0% to 30.8% for the 7T 8‐channel coil, and from 91.1% to 50.9% for the 10.5T 16‐channel coil when compared to conventional methods using 50,000 random RF shims.

**Conclusion:**

The developed $B_{1}^{+}$‐driven RF co‐simulation optimization method in this work can be used to generate a more accurate EM simulation model of custom‐built arrays and potentially reduce the need for large safety margins, with improved RF power management.

## 1 Introduction

Magnetic Resonance Imaging technology is a rapidly growing area of scientific research. Today, MRI systems are a crucial tool for medical diagnosis and surgical planning, with a continuing demand for improved Signal‐to‐Noise Ratio (SNR) in images. This objective can be achieved by developing UHF MRI systems, leveraging the relationship between SNR and the static magnetic field strength $B_{0}$ [1]. Driven by the expected increase in SNR and shorter acquisition times, higher spatial resolution, and improved temporal resolution (e.g., fMRI), RF technology development for UHF MRI applications has seen an increasing focus in medical imaging research. This trend has introduced new engineering challenges for the design of MRI RF coils [2]. Since the Larmor frequency increases with $B_{0}$, $\omega =\gamma B_{0}$, where γ is the gyromagnetic ratio for hydrogen nuclei, the RF coil designs have become more complicated due to the high‐frequency effects. With the Food and Drug Administration (FDA) approval of Siemens MAGNETOM Terra with pTx capability, multichannel transmit arrays are becoming more frequently used at 7T and beyond, providing more homogenous $B_{1}^{+}$ field and flip angle distributions at these frequencies. However, there are multiple engineering challenges, including addressing the RF safety concerns, maximizing the $B_{1}^{+}$ efficiency, minimizing the radiation losses, and improving the decoupling between elements [3, 4, 5, 6]. These challenges can be met through utilization of fast and rigorous 3D full‐wave EM simulation tools for RF coils [7].

EM simulations are widely used for designing and evaluating UHF RF coils. In most cases, RF coils include a large number of lumped elements to tune and decouple the array's elements, in addition to the matching networks. The typical approach is to define each lumped element as an internal RF port within the 3D full‐wave simulation domain in order to allow these lumped elements to be tuned in the RF co‐simulation environment [8, 9, 10, 11]. The rationale behind this approach is that the co‐simulation is a lightweight computation in comparison to the 3D full‐wave simulations. The EM simulations are often computationally expensive in order to repeatedly optimize or fine‐tune lumped elements treated as fixed boundary conditions within the 3D geometry. On the other hand, the co‐simulation is simply an RF circuit evaluation that can be performed inside an optimization program to obtain the desired performance in a reasonable time frame. The co‐simulation is performed for different purposes, including providing guidance for the design of the matching and tuning circuits, as well as optimizing the S‐parameters of the RF coil, which is an important aspect of every RF coil design. Throughout the MRI literature, this method is commonly used for both designing and tuning an RF coil to match the fabricated designs. However, inside the MRI scanner environment, there are plenty of factors that can affect the S‐parameters and EM fields, including the magnet bore, the patient's table, losses in the RF transmit cables and the RF coil connection interfaces to the MRI system, and tolerances of the lumped elements values. For these reasons, developing an RF coil simulation model that accurately predicts the resulting fields inside the MR scanner becomes increasingly complicated as we push for higher frequencies.

Several commercial software packages provide functionalities for RF circuit analysis, simultaneous external RF excitation, and EM field combination. Among them, ANSYS HFSS, Simulia CST Studio, and Sim4Life are commonly used for MRI applications. Open‐source packages such as CoSimPy are also widely used in the MRI community [12]. However, these built‐in functionalities often lack the flexibility required to easily tweak or explore new optimization objectives or techniques. Moreover, 3D full‐wave EM solver software typically combine the EM fields across the entire simulation domain, which can be extremely slow to practically run a field‐based optimization. As a result, researchers often develop their own tools for greater flexibility and efficiency. In the MRI literature, one can find examples of studies that attempted to accelerate EM/RF circuit co‐simulations using closed‐form approximations tailored to specific RF coils types with a lot of limitations [13]. Others have proposed customized formulations for particular design problems often demonstrated with an optimization [14, 15, 16, 17]. Also, there are open‐source packages for general‐purpose RF coil co‐simulations, but these options restrict the user to a specific programming platform and may lack the flexibility to be adapted to one's needs [12]. Moreover, inaccuracies in S‐parameters calculations and combined fields results have been noticed by researchers when using these implementations. Despite the apparent simplicity of the problem and the wide availability of such solutions, there remains a tangible need for a straightforward yet complete formulation specifically targeting MRI RF coil designs. Such a formulation would enable researchers to develop their own in‐house routines for EM fields and RF circuit co‐simulations on any suitable programming environment. The true advantage of such a technique would be the ability to export EM fields on fixed slices within the 3D simulation domain with the desired resolution. In this case, the computations associated with combining the EM fields due to external simultaneous excitation are dramatically reduced. For example, one can restrict the calculations to the desired field result, for example, $B_{1}^{+}$ only, and optimize the simulation model to obtain a desired $B_{1}^{+}$ distribution, in contrast to evaluating all field results every time.

In this work, a customized RF co‐simulation method for multichannel RF coils is presented. The proposed method enables rapid calculation of various RF circuit quantities such as the S‐matrix, incident and accepted powers, and voltages and currents at the RF ports. In addition, it allows for fast evaluation of simulated $B_{1}^{+}$ maps, thereby enabling optimization of lumped elements, phase shifters, and attenuators to minimize the error between simulated and experimentally measured $B_{1}^{+}$ maps.

## 2 Methods

### 2.1 General Formulation

The problem of evaluating the EM fields and RF circuit co‐simulation is illustrated in Figure 1. The 3D EM problem is described by N internal RF ports (full‐wave solver space) where each RF port can be connected to an external RF port or lumped element terminations in the co‐simulation. Initially, all RF ports are assumed to be connected to arbitrary 2‐port matching networks $M_{1},\ldots ,M_{N}$. In general, the co‐simulation network can be a separate 2N×2N device connecting all internal and external RF ports. Although this is a more general scenario, this choice does not allow for a fixed RF network formulation. The only approach to solve the most general scenario is the signal flow graph technique, which requires a more complex solution and programming paradigm [18, 19]. Fortunately, for the vast majority of RF coils designs, the typical RF network consists of external RF ports or lumped element terminations directly applied to the EM simulation internal RF ports. In addition to that, one can include external matching networks between the external RF ports and the corresponding internal ones for further tuning. This scenario allows for a closed‐form expression for the co‐simulation network, which provides an easier implementation. Also, this scenario results in faster fields computations, allowing for fields‐based optimizations as demonstrated later in this work. The 3D full‐wave EM simulation can be performed on any simulation software, and it should provide EM field quantities, such as E‐field and H‐field, in addition to the S‐matrix at the internal RF ports reference plane

(1) $$S=\left[\begin{array}{lll} S_{1,1} & \ldots & S_{1,N}\\ \vdots & \ddots & \\ S_{N,1} & & S_{N,N} \end{array}\right].$$

The voltages and currents on each internal RF port can be expressed as

(2) $$V=\left[\begin{array}{l} V_{1}\\ \vdots \\ V_{N} \end{array}\right],\;\text{and}\;I=\left[\begin{array}{l} I_{1}\\ \vdots \\ I_{N} \end{array}\right].$$

**FIGURE 1:**
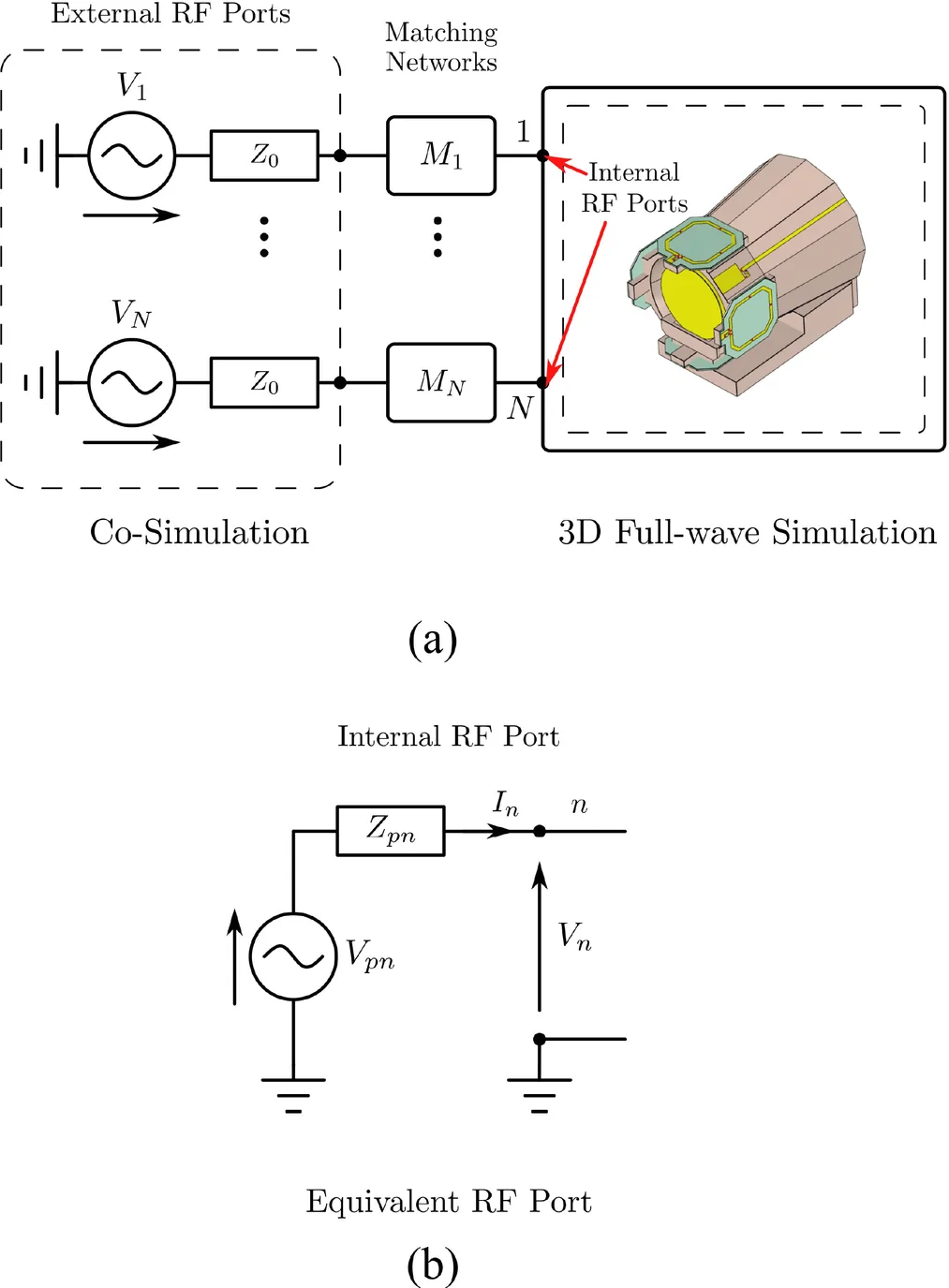
An illustration of a typical 3D full‐wave EM simulation of an MRI RF coil with N internal RF ports (EM simulation RF ports). (a) The co‐simulation represents an external excitation network described by N external RF ports and N arbitrary matching networks. (b) The equivalent circuit at the internal RF port n, where 1≤n≤N. The equivalent excitation voltage is $V_{pn}$, and the complex impedance is $Z_{pn}$. All external RF ports are assumed to have internal real impedances $Z_{0}$, typically 50Ω.

It is recommended to keep the polarity definitions consistent. Through this work, all circuit and field quantities are treated as maximum values (peak values) for clarity. The equivalent Z‐matrix for the EM simulation at the internal RF ports is readily found by

(3) $$Z=\left[U_{N}+S\right]\cdot {\left[U_{N}-S\right]}^{-1}\cdot Z_{0},$$

where $U_{N}$ is a unity identity matrix of size N×N and |Z|≠0 allowing for the definition V=Z·I. The $Z_{0}$ corresponds to the system's reference impedance, which is typically 50Ω. In general, RF systems can have arbitrary complex reference impedances, which requires power‐waves analysis beyond the scope of this work [20]. Here, one can define arbitrary RF ports excitation and complex ports impedances as

(4) $$V_{p}=\left[\begin{array}{l} V_{p1}\\ \vdots \\ V_{pN} \end{array}\right],\;\text{and}\;Z_{p}=\left[\begin{array}{lll} Z_{p1} & & \\ & \ddots & \\ & & Z_{pN} \end{array}\right].$$

Note that $Z_{p}$ is a diagonal matrix based on the definition in Figure 1a. The coupling between the RF ports only takes place inside the 3D full‐wave simulation domain. Now, imposing the arbitrary ports excitation and complex impedances from Figure 1b gives the relation

(5) $$\left[U_{N}+Z_{p}\cdot Z^{-1}\right]\cdot V=V_{p},$$

which is subject to the restriction $|U_{N}+Z_{p}\cdot Z^{-1}|\neq 0$, unless someone decided to force $Z_{p}=-Z$ which renders $V=V_{p}=0$. It is worth mentioning that the scenario of $\left[U_{N}+Z_{p}\cdot Z^{-1}\right]\cdot V=0$ contains information about the resonance of the structure. Besides this unusual case, one can easily obtain

(6) $$\begin{array}{cc} V & ={\left[U_{N}+Z_{p}\cdot Z^{-1}\right]}^{-1}\cdot V_{p},\;\text{and}\\ I & ={\left[Z+Z_{p}\right]}^{-1}\cdot V_{p}. \end{array}$$

The incident RF powers can be directly calculated as

(7) $$P_{\text{inc}}=\frac{1}{8}{\left[ℛe\left\{Z_{p}\right\}\right]}^{-1}\cdot |V_{p}{|}^{2},$$

where $|V_{p}{|}^{2}={\left[|V_{p1}{|}^{2},\ldots ,|V_{pN}{|}^{2}\right]}^{T}$, and T denotes the transpose operation, and the total incident RF power is simply given by

(8) $$P_{\text{inc}}=\overset{N}{\underset{n=1}{\sum }}P_{\text{inc},n}.$$

Similarly, the accepted powers can be calculated by

(9) $$P_{\text{acc}}=\frac{1}{2}ℛe\left\{I^{H}\cdot V\right\},$$

where H denotes a Hermitian transpose, and the total accepted power is

(10) $$P_{\text{acc}}=\overset{N}{\underset{n=1}{\sum }}P_{\text{acc},n}.$$

The above formulation will be utilized in the following sections to evaluate different aspects of the co‐simulation problem, where the $V_{p}$ and $Z_{p}$ definitions will be changed accordingly. Repeating the above with $\tilde{S}$ that represents the external RF ports network gives the incident and accepts powers at the external RF ports reference plan.

### 2.2 The Overall S‐Matrix Calculation

An important calculation from the co‐simulation is the evaluation of the overall S‐matrix for the entire system, including the lumped elements, matching networks, and the external RF ports effects. First, the number of the external RF ports $N_{e}$ is assumed to be $N_{e}\leq N$, as mentioned earlier. For each internal RF port n, there will be a 2×2 network represented by the S‐matrix

(11) $$S^{\left(n\right)}=\left[\begin{array}{ll} S_{1,1}^{\left(n\right)} & S_{1,2}^{\left(n\right)}\\ S_{2,1}^{\left(n\right)} & S_{2,2}^{\left(n\right)} \end{array}\right],$$

where 1≤n≤N. If a matching network is applied, one can directly cascade any configuration of shunt and series lumped elements using the star product (⋆) defined in Appendix A. For example, a matching network consisting of k arbitrary lumped elements has an S‐matrix $S^{\left(n\right)}=S_{1}^{\left(n\right)}⋆S_{2}^{\left(n\right)}⋆\ldots ⋆S_{k}^{\left(n\right)}$. For a series impedance Z and a shunt admittance Y, lumped elements that are commonly used in matching networks, one can use the expressions in Equations (A6) and (A7), respectively.

This unified representation approach makes the formulation easier to implement, as seen in the next steps. Note that applying an ideal short‐circuit on an internal RF port is also possible to achieve by using a reflection coefficient of −1; however, allowing an ideal open‐circuit (a reflection coefficient of +1) on a termination RF port will result in an overflow in the field combination procedure provided in the next section. Thus, for a practical implementation, it is recommended to restrict the load terminations to $0<|Z_{n}|<\infty$ in order to avoid any numerical issues. On the other hand, if no matching network is desired, one can simply use $S^{\left(n\right)}=J_{2}$ between the internal and external RF ports, where $J_{2}$ is the backward identity matrix. Once all matching networks and terminations calculations are computed at a given frequency, the overall S‐matrix for the system is directly achieved by [18].

(12) $$\tilde{S}=W_{ba}+W_{bx}\cdot {\left[U_{4N}-W_{xx}\right]}^{-1}\cdot W_{xa},$$

where

(13) $$W_{xx}=\left[\begin{array}{llll} 0_{N} & 0_{N} & 0_{N} & U_{N}\\ \Gamma_{2,2} & 0_{N} & 0_{N} & 0_{N}\\ 0_{N} & U_{N} & 0_{N} & 0_{N}\\ 0_{N} & 0_{N} & S & 0_{N} \end{array}\right],$$

(14) $$W_{bx}=\left[\begin{array}{llll} \Gamma_{1,2} & 0_{N} & 0_{N} & 0_{N} \end{array}\right],$$

(15) $$W_{xa}=\left[\begin{array}{l} 0_{N}\\ \Gamma_{2,1}\\ 0_{N}\\ 0_{N} \end{array}\right],\;W_{ba}=\left[\begin{array}{l} \Gamma_{1,1} \end{array}\right],$$

and

(16) $$\Gamma_{i,j}=\left[\begin{array}{lll} S_{i,j}^{\left(1\right)} & & \\ & \ddots & \\ & & S_{i,j}^{\left(N\right)} \end{array}\right].$$

The system in Equation (12) simplifies into

(17) $$\tilde{S}=\Gamma_{1,1}+\Gamma_{1,2}\cdot S\cdot {\left[U_{N}-\Gamma_{2,2}\cdot S\right]}^{-1}\cdot \Gamma_{2,1}.$$

Note that in the above, $0_{N}$ is a null matrix of size N×N. Since the resulting S‐matrix is of size N×N, for $N_{e}$ external RF ports, the final overall S‐matrix becomes $\tilde{S}:={\tilde{S}}_{1:N_{e},1:N_{e}}$. The above system is numerically stable regardless of the co‐simulation network choices. It is recommended to use the expression in Equation (17) since it results in N×N matrices operations instead of 4N×4N as in Equation (12).

### 2.3 The Combined Fields Calculation

The evaluation of the combined fields due to an arbitrary simultaneous external RF excitation is another important calculation for MRI RF coils. This allows for the calculation of the EM fields while properly taking into account all the effects from the co‐simulation network. Here, $V_{p}$ and $Z_{p}$ matrices will be redefined as mentioned earlier. Assuming an external excitation vector (RF shim) from the $N_{e}$ external RF ports, one can define

(18) $$V_{p}^{0}=\left[\begin{array}{l} V_{p1}^{0}\\ \vdots \\ V_{pN}^{0} \end{array}\right].$$

In the above, for $n>N_{e}$, $V_{p}^{0}$ vector is padded with zeros, that is, $V_{pn}^{0}=0$ for $(N-N_{e})<n\leq N$. Notice that $V_{pn}^{0}$ is the voltage of the voltage source n, not the available voltage from the source. Next, in order to account for the matching networks, an additional step is needed. For the internal RF port $n\leq N_{e}$, ${\hat{S}}^{\left(n\right)}$ is defined as ${\hat{S}}^{\left(n\right)}=S_{0}^{\left(n\right)}⋆S^{\left(n\right)}$, where $S_{0}^{\left(n\right)}$ is a series $Z_{0}$ impedance S‐matrix which reduces to

(19) $$S_{0}^{\left(n\right)}=\frac{1}{3}\left[\begin{array}{ll} 1 & 2\\ 2 & 1 \end{array}\right].$$

For other internal RF ports that are not connected to an external RF port, that is, $(N-N_{e})<n\leq N$, ${\hat{S}}^{\left(n\right)}$ is kept as ${\hat{S}}^{\left(n\right)}=S^{\left(n\right)}$ from Equation (A8). Thus, $V_{p}$ and $Z_{p}$ elements are redefined as follows

(20) $$Z_{pn}=Z_{0n}\frac{[1+{Ŝ}_{1,1}^{\left(n\right)}][1+{Ŝ}_{2,2}^{\left(n\right)}]-{Ŝ}_{1,2}^{\left(n\right)}{Ŝ}_{2,1}^{\left(n\right)}}{[1+{Ŝ}_{1,1}^{\left(n\right)}][1-{Ŝ}_{2,2}^{\left(n\right)}]+{Ŝ}_{1,2}^{\left(n\right)}{Ŝ}_{2,1}^{\left(n\right)}},$$

and

(21) $$V_{pn}=V_{pn}^{0}\frac{2{Ŝ}_{2,1}^{\left(n\right)}}{[1+{Ŝ}_{1,1}^{\left(n\right)}][1-{Ŝ}_{2,2}^{\left(n\right)}]+{Ŝ}_{1,2}^{\left(n\right)}{Ŝ}_{2,1}^{\left(n\right)}}.$$

The above‐described expressions guarantee numerical stability for any arbitrary configuration with the choice $|Z_{n}|<\infty$ for all impedances. In order to export the EM fields from the 3D full‐wave simulation corresponding to an internal RF port n, one must apply an excitation directly on the internal RF port n while terminating all other ports with $Z_{0}$ and then evaluate the resulting fields. Fortunately, this is the standard approach used in almost all EM simulation tools. Usually, a fixed voltage or a fixed power is applied in each internal RF port individually, and the results are stored as $E_{n}$ and $H_{n}$. The following algorithm should work regardless of the default settings, but it is recommended to identify the default internal excitation applied by EM simulation tools. For simplicity, a fixed voltage of 1V will be assumed when computing the EM fields. For a specific RF shim on the external RF ports, one can calculate V from Equations (6), where $Z_{p}$ and $V_{p}$ are calculated from (20) and (21), respectively. Based on this calculation, the scaling ratios for the EM fields are calculated by

(22) $$\alpha ={\left[U_{N}+Z_{0}\cdot Z^{-1}\right]}^{T}\cdot {\left[U_{N}+Z_{p}\cdot Z^{-1}\right]}^{-1}\cdot V_{p}.$$

Thus, the combined fields at any given spatial position r in the space within the EM simulation domain is found as

(23) $$E(r)=\overset{N}{\underset{n=1}{\sum }}\alpha_{n}E_{n}(r),\;\text{and}\;H(r)=\overset{N}{\underset{n=1}{\sum }}\alpha_{n}H_{n}(r).$$

The above expressions can be used for other EM fields components such as volume and surface current densities where the superposition principle holds.

### 2.4 Optimization Examples

#### 2.4.1 General Description

The formulation provided in the previous sections will be demonstrated on an MRI RF coil optimization problem. The goal is to show a practical example where this formulation can be used to improve the accuracy of RF coil modeling against other common techniques often used in MRI literature. Two RF coils were considered for this demonstration at 7T and 10.5T, as illustrated in Figure 2. The 8‐channel pTx knee coil illustrated in Figure 2a was developed and fabricated for a 7T MRI system. An EM simulation model was generated for this coil design in CST Studio (Darmstadt, Germany), as shown in Figure 2b. The coil was 3D‐printed using Polycarbonate (PC) filament (Atomic Filament, Kendallville, IN), and the metallic traces were created from a copper tape of width 5 mm. The loops were fabricated on thin circuit boards, while the monopole elements were applied directly to the coil holder with the details given in Figure 2c,d. Each loop includes 3 tuning capacitors and 4 additional capacitors to match the elements at 297MHz (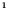
H (proton) Larmor frequency at 7T). Similarly, each monopole includes an inductor and a capacitor for tuning and matching at 297MHz. Moreover, coaxial cables were applied to the 8 RF ports of the coil in addition to cable traps to minimize common‐mode currents on the coaxial cables shielding. A cylindrical phantom was placed inside the coil and positioned accurately at the center using a 3D‐printed holder. The cylindrical phantom was made of Polyvinylpyrrolidone (PVP) gel with electrical properties of $\epsilon_{r}=50.98$, σ=0.58 (S/m), and a mass density ρ=1000 (kg/m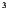) at 297MHz. The diameter of the cylinder is 116 mm and the height of the cylinder is 200 mm. The phantom holder was fabricated using a PC 3D printer filament. The fabricated coil was tuned and matched to a human knee‐shaped phantom filled with a similar PVP solution.

**FIGURE 2:**
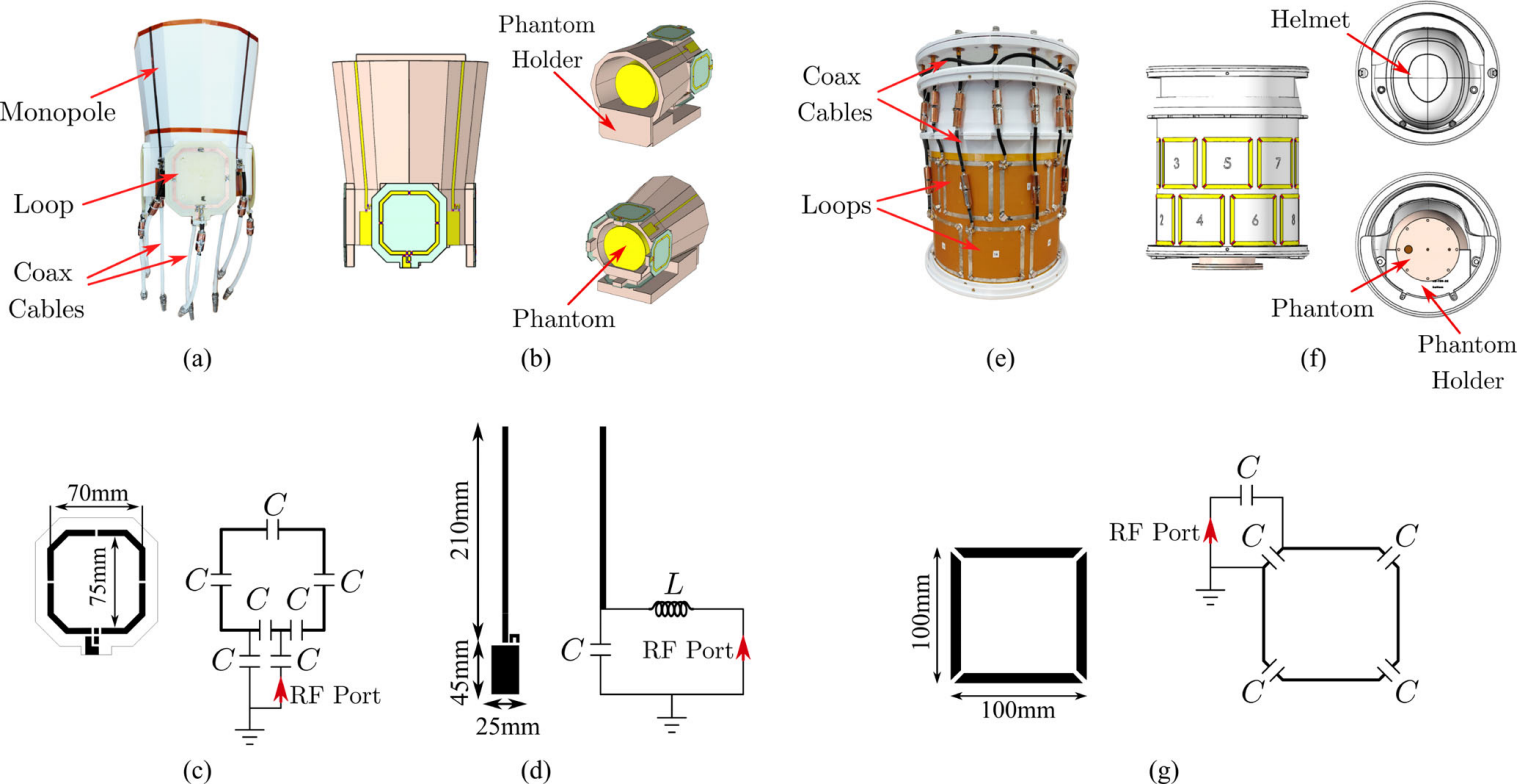
An illustration of the RF coils used in this study. The 7T knee coil array (8 channels) is made of 4 monopole‐like radiators and 4 loops: (a) The fabricated RF coil picture. (b) The simulation model built in CST Studio. (c) and (d) are depictions of the loops and monopoles elements, respectively. The 10.5T head coil array (16 channels) is made of 16 identical loops: (e) The fabricated RF coil picture. (f) The simulation model built in CST Studio. (g) A depiction of the loops elements.

The 16‐channel pTx head coil illustrated in Figure 2e was developed and fabricated for a 10.5T MRI system [21]. An EM simulation model was generated for this coil design also on CST Studio, as shown in Figure 2f. The coil body and helmet were 3D‐printed using Polyethylene Terephthalate Glycol (PETG) filament by Atomic Filament (Kendallville, IN). The loops details are given in Figure 2g. Each loop includes 4 tuning capacitors and 1 matching capacitor to match the elements at 447MHz (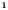
H (proton) Larmor frequency at 10.5T). Moreover, extension coaxial cables were applied to the 16 RF ports of the coil with equal cable lengths of 20cm. A head‐shaped phantom was placed inside the coil and positioned accurately using a 3D‐printed PC holder in order to tune and match the fabricated coil. The head phantom was made of PVP gel with electrical properties of $\epsilon_{r}=49.24$, σ=0.67 (S/m), and a mass density ρ=1120 (kg/m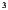) at 447MHz. The fabricated coil was tuned and matched to the phantom at 447MHz. For the head coil, the bore shield used in the MR scanner was implemented in the EM simulations using a cylinder of diameter 640 mm for better accuracy.

#### 2.4.2 Optimization Setups

The optimization setups for the two RF coils presented in the previous section are given in Figure 3. In order to allow for more flexibility in optimization, all lumped elements were treated as independent variables that can be tuned within the co‐simulation as shown in Figure 3. Moreover, additional ideal phase shifters and attenuators were placed before the external RF ports in order to fine tune the models, taking into account any nonequal cables lengths and other effects. Additionally, for the 10.5T head coil, matching networks were placed in front of each external RF port mimicking the fabricated coil design.

**FIGURE 3:**
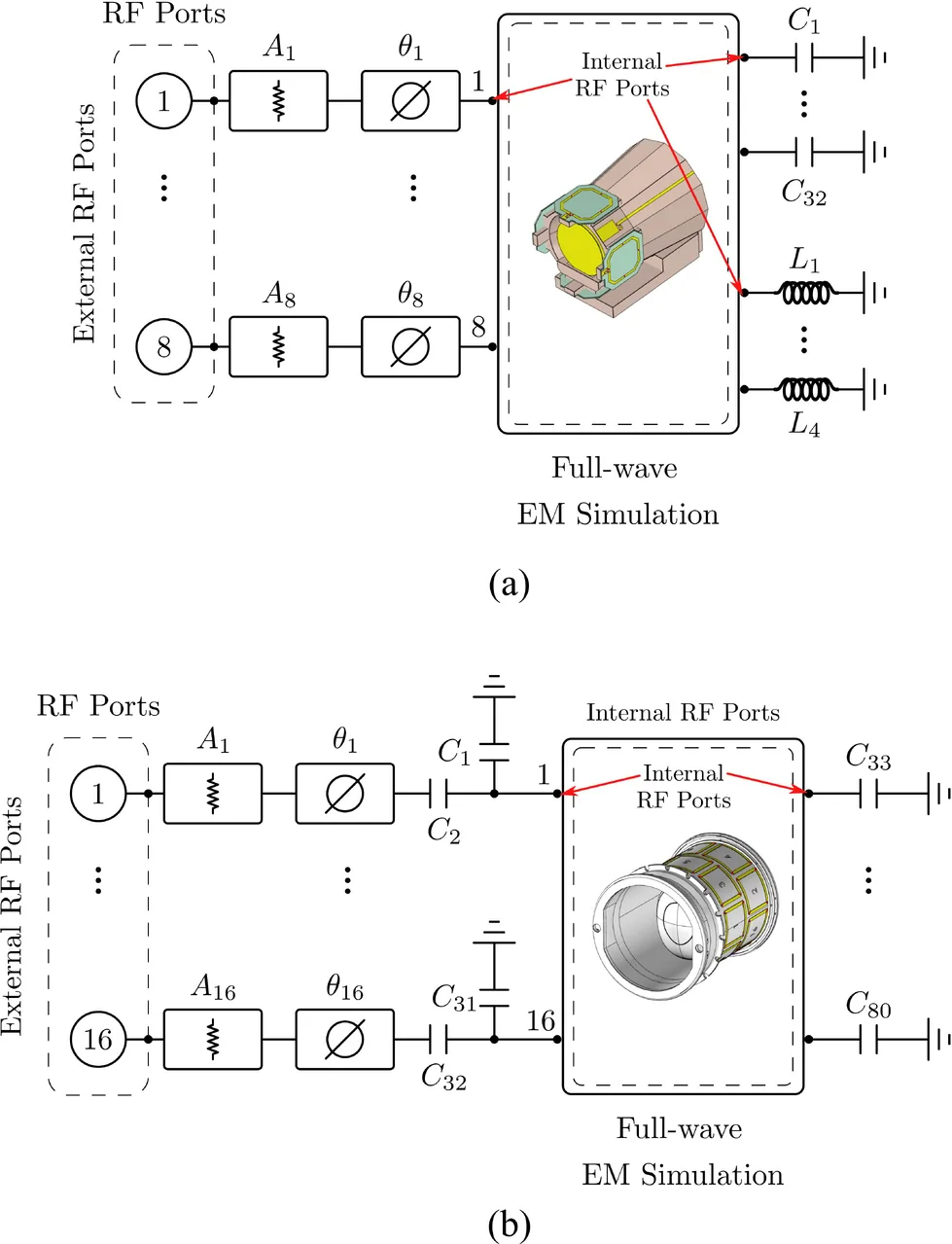
The RF co‐simulation schematic of the 7T knee and 10.5T head coil arrays with all lumped elements placed outside the 3D full‐wave EM simulation models. (a) The 7T knee coil includes 8 RF ports representing the external RF excitation, 32 RF ports for the lumped capacitors, and 4 RF ports for the lumped inductors, totaling 44 internal RF ports. (b) The 10.5T head coil includes 16 RF ports representing the external RF excitation and 48 RF ports for the lumped capacitors, totaling 64 internal RF ports. For both designs, additional attenuators and phase shifters were added to the external RF ports.

In order to demonstrate the advantage of the developed formulation in this work on pTx arrays, two methods were considered to optimize the RF co‐simulation tunable variables. The first method (proposed method) relies on the measured $B_{1}^{+}$ fields to tune the simulation model via the co‐simulation combined fields. For the sake of clarity, this method will be called a $B_{1}^{+}$‐driven method. This method is proposed as a superior method compared to those reported in the MRI literature. The $B_{1}^{+}$‐driven method requires the acquisition of relative $B_{1}^{+}$ maps which can be obtained using a 2D GRE pulse sequence [22] (TE = 2.2 ms, TR = 100 ms and flip angles of 20deg and 15deg for 7T and 10.5T, respectively) in addition to absolute $B_{1}^{+}$ maps which were obtained by using an actual flip angle (AFI) pulse sequence [23]. The use of relative $B_{1}^{+}$ maps was necessary to evaluate the accuracy of two approaches in a comprehensive manner. The synthesis of arbitrary RF shims from relative $B_{1}^{+}$ mapping individual channels is a widely used approach in the literature. The measured relative $B_{1}^{+}$ maps were acquired at equally spaced slices, while the simulated EM fields results were exported individually for each internal RF port on the same slices in order to establish a comparison. An optimization program was developed on MATLAB by MathWorks (Massachusetts, United States) in order to minimize a typical normalized root mean square error (NRMSE) between the co‐simulated and measured $B_{1}^{+}$ maps. Both absolute maps (measured and co‐simulated) were obtained by using random shims in addition to Circular Polarization (CP), Zero Phase (ZP), and channel‐wise shims. The 7T knee and the 10.5T head coils $B_{1}^{+}$‐fields were compared to measurements using 20 and 22 RF shims, respectively. For the knee and head coils, 8 and 16 shims out of the 20 and 22 RF shims were channel‐wise, respectively. The optimization goal was set to reduce the NRMSE between the measured and co‐simulated $B_{1}^{+}$‐fields on all the RF shims stated above. The cost function for the $B_{1}^{+}$‐driven method is given as follows

(24) $$f=\frac{1}{N_{\varphi }}\overset{N_{s}}{\underset{s=1}{\sum }}\overset{N_{\varphi }}{\underset{\varphi =1}{\sum }}{\text{NRMSE}}_{s,\varphi }\left({\left|B_{1}^{+}\right|}^{\text{Meas}},{\left|B_{1}^{+}\right|}^{\text{CoSim}}\right),$$

where $N_{s}$ and $N_{\varphi }$ are the number of slices and shims, respectively.

The second method is a typical approach commonly found in the literature [24], where the optimization goal is to match the RF co‐simulated S‐matrix to the measured ones. For the sake of clarity, this method will be called an S‐matrix‐driven method. The cost function is typically expressed as

(25) $$f=\frac{1}{N_{e}^{2}}\overset{N_{e}}{\underset{i=1}{\sum }}\overset{N_{e}}{\underset{j=1}{\sum }}{\left|S_{i,j}^{\text{Meas}}-S_{i,j}^{\text{CoSim}}\right|}^{2}.$$

The cost function above compares all the S‐matrix elements, while in the literature only the neighboring elements are usually considered [25]. According to initial trials, including all the S‐matrix elements produced better overall results. Also, since the couplings between far‐elements are small, this calculation does not slow down the performance nor deviate the cost function from its intended objectives. The tuning variables for the S‐matrix‐driven method were set initially according to the fabricated device nominal values and were allowed to optimize in a wide range. The RF co‐simulation method developed in Section 2.2 was used for the purpose of S‐matrix optimization. One should note that the same optimization can also be performed using any third‐party software. The total number of tunable variables for the 7T knee coil and the 10.5T head coil were 52 and 112, respectively.

A graphical illustration for the two methods workflows is given in Figure 4. For both methods a MATLAB built‐in derivative‐free pattern search algorithm patternsearch was utilized to perform the optimizations [26]. For both optimization methods, an initial optimization starting from the nominal values was performed in order to maximize the power delivery into all elements with the objective of matching all the external RF ports. Then, the results from this initial optimization were used as a starting point for both $B_{1}^{+}$‐driven and S‐matrix‐driven methods. For the stopping criteria, the optimizations were repeated multiple times until saturation, that is, little to no additional improvements. Once the optimizations were finished, a total of 50,000 random magnitude and phase RF shims were applied to the two resulting optimized simulation models: viz., the $B_{1}^{+}$‐driven and the S‐matrix‐driven methods, and the resulting NRMSE of $B_{1}^{+}$ against measurements were evaluated to generate a comprehensive statistical comparison. The error evaluation was performed on all slices obtained from experimental measurements and simulations for each RF shim individually.

**FIGURE 4:**
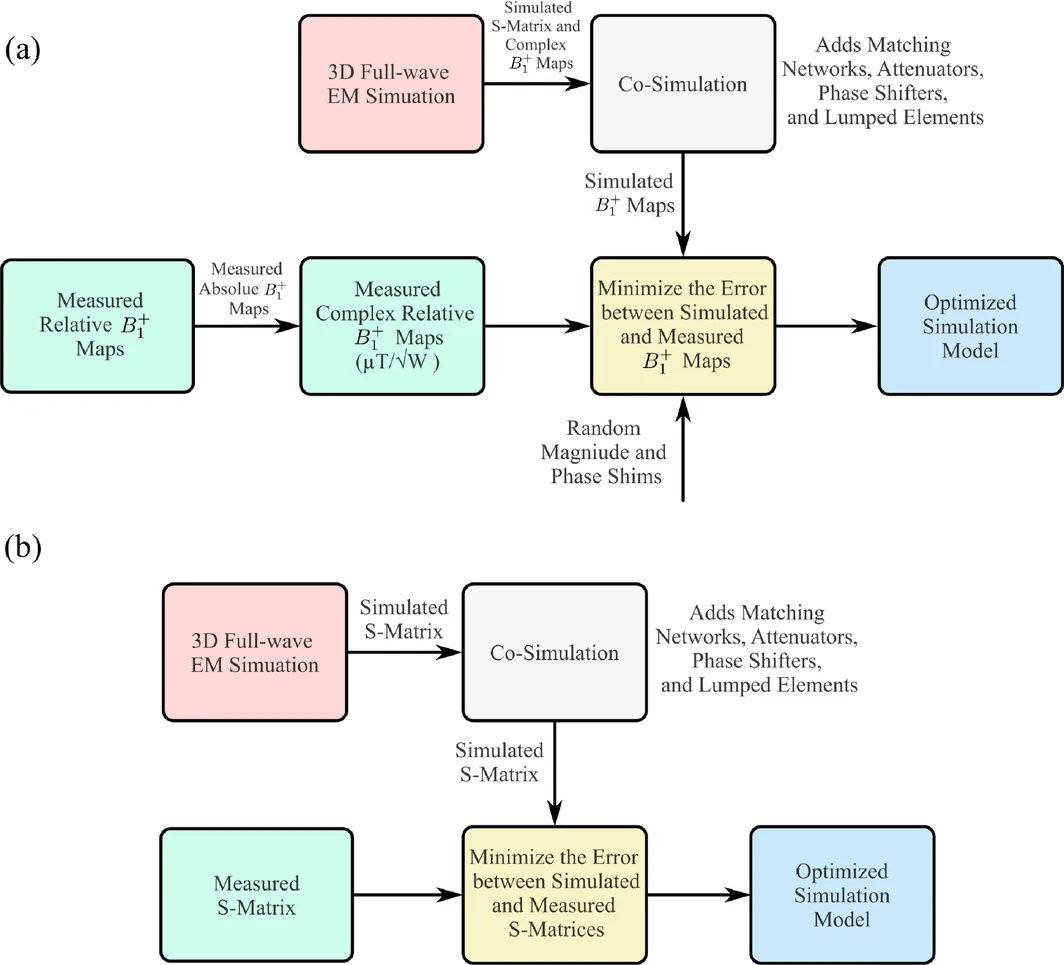
A graphical illustration of the two optimization method workflows used to tune the RF coils simulation models. (a) The $B_{1}^{+}$‐driven method. (b) The S‐matrix‐driven method.

### 2.5 SAR Validation

In addition to the optimization examples described in the previous section, a validation experiment was conducted to evaluate the accuracy of the predicted SAR levels and patterns using the $B_{1}^{+}$ and S‐matrix optimized models. Here, the 7T knee coil was considered to measure the 10g average SAR inside a blunted cone‐shaped phantom, as shown in Figure 10. Two central orthogonal vertical plane cuts were considered (against x and y) corresponding to sagittal and coronal cuts inside the phantom. The measurements were performed using a $B_{0}$‐free in‐house developed setup consisting of a 3‐axis motor system that controls the position of a SAR probe in 3D space, allowing for volumetric data sets acquisition, all inside a Faraday cage [27, 28, 29]. The phantom was filled with PVP with a dielectric constant of 54.9, electrical conductivity of 0.89 (S/m), and mass density of 1045 (kg/m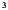). For each measurement cut, the data were acquired using a DAKS12 SAR probe from SPEAG (Zurich, Switzerland) on two parallel planes with a transverse resolution of 8 mm × 8 mm and a gap of 16.61 mm between the two planes. SAR was then averaged within a 24 mm × 24 mm × 16.61 mm volume, yielding approximately 10g of PVP. Three random phases were evaluated in this setup and compared to both optimized models from the S‐matrix and $B_{1}^{+}$‐driven methods.

## 3 Results

Figure 5 illustrates the magnitude of S‐matrices obtained from the S‐matrix‐driven method compared to the experimentally measured S‐matrices for the 7T and 10.5T RF coils. A similar comparison for the phases is provided in Figure S1. The S‐matrix RMSE resulted in 0.005 and 0.0085 for the knee and the head coils using the error definition in Equation (25), respectively. Figure 6 shows the 7T knee coil channel‐wise $B_{1}^{+}$ fields comparisons on 2 slices obtained by the $B_{1}^{+}$‐driven and S‐matrix‐driven methods. Similar comparisons for the 10.5T head coil on 3 slices obtained by the $B_{1}^{+}$‐driven and S‐matrix‐driven methods are shown in Figures S2 and S3, respectively. Figure 7 shows samples for $B_{1}^{+}$ fields due to arbitrary shims for the 7T knee coil while comparing the outcome from the $B_{1}^{+}$‐driven and the S‐matrix‐driven methods. In Figure 7, shim #1 is CP, shim #2 is ZP, and shims #3 to #6 are random magnitude and phase shims.

**FIGURE 5:**
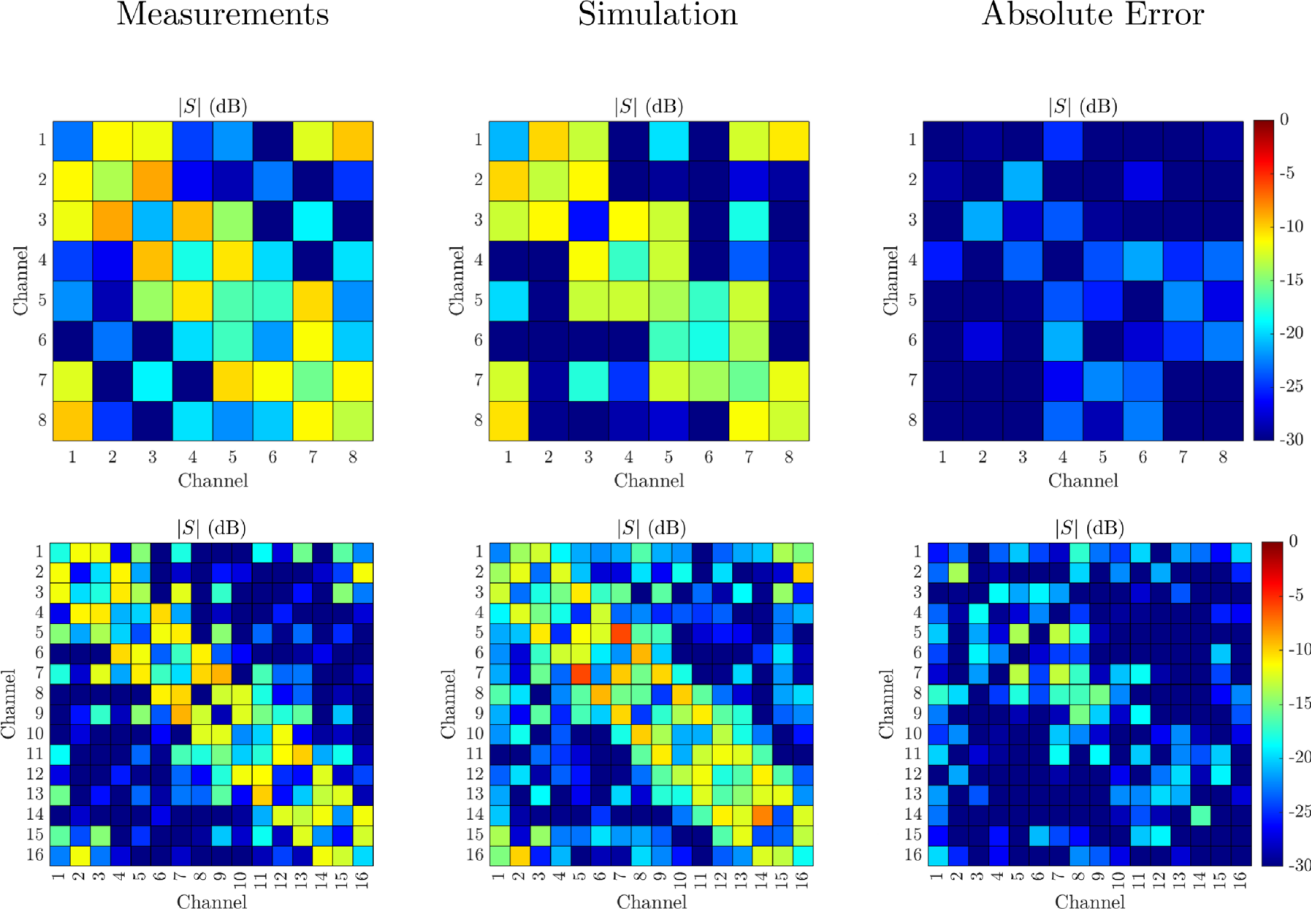
The optimized S‐matrix magnitudes for the S‐matrix‐driven method compared to the measured S‐matrix for the 7T knee coil and 10.5T head coil illustrated on rows 1 and 2, respectively. The first column is the measured S‐matrix data. The second column is an optimized co‐simulated S‐matrix. The last column is the absolute error between the measured and co‐simulated S‐matrix, all are presented in dB scale.

**FIGURE 6:**
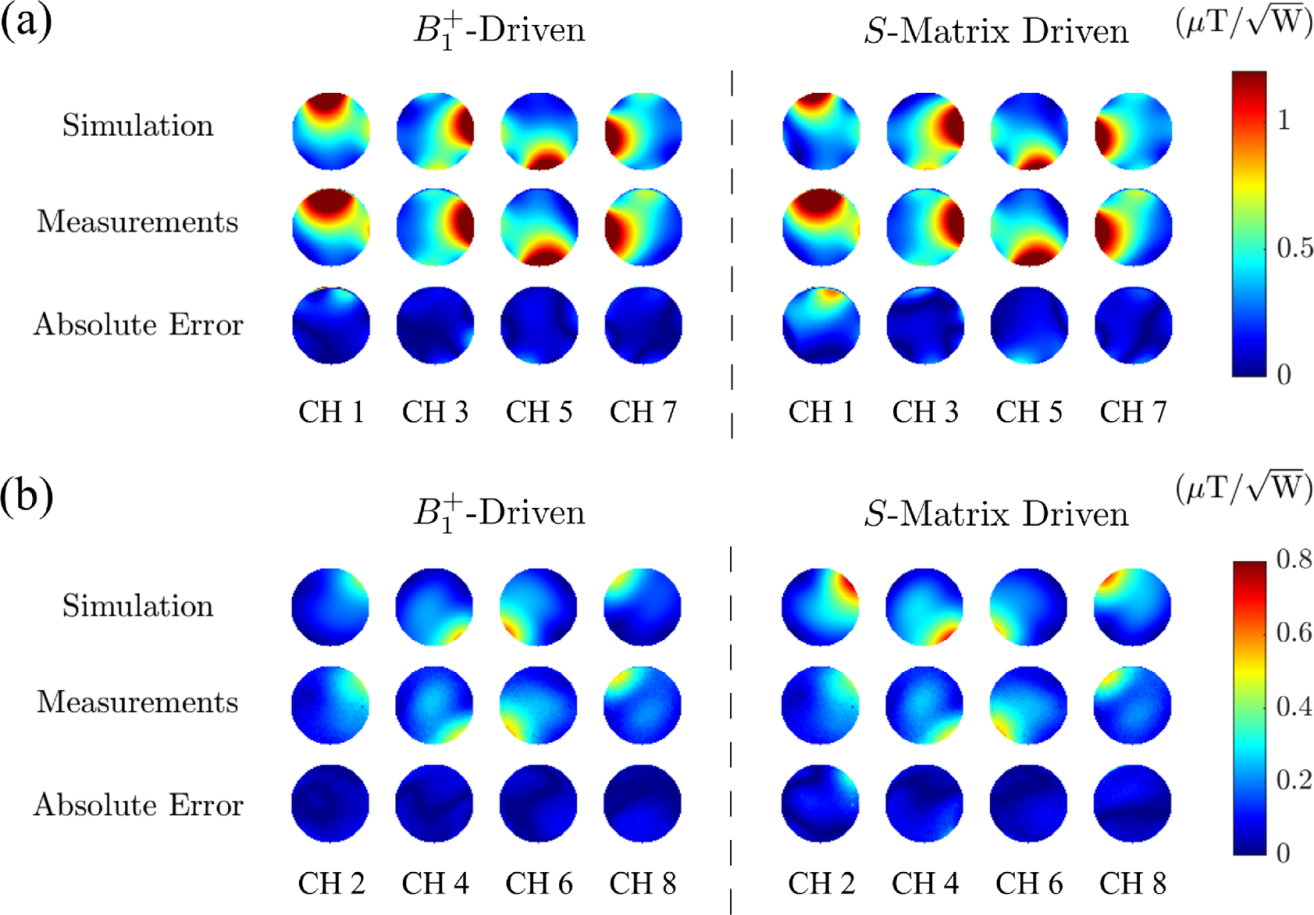
The resulting channel‐wise $B_{1}^{+}$ maps from each optimization method on slices inside the phantom compared to the measurements for the 7T knee coil. The comparisons were made on slices close to the center of each element type. (a) The loops elements (Channels 1, 3, 5, and 7). (b) The monopoles elements (Channels 2, 4, 6, and 8). Within each subfigure, the left and right columns are the results for the $B_{1}^{+}$‐driven and the S‐matrix‐driven methods. The absolute error results were included for a visual comparison.

**FIGURE 7:**
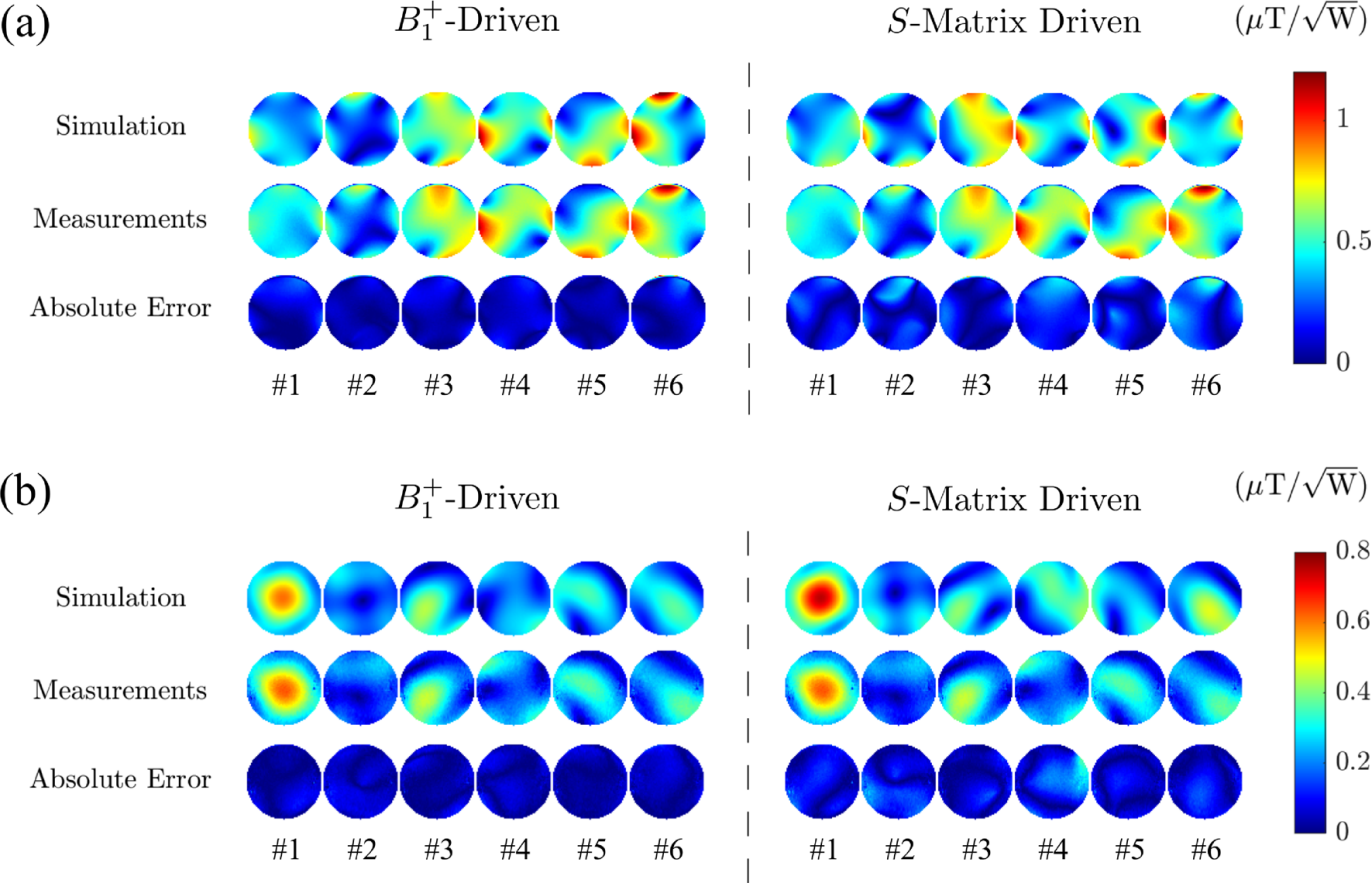
Sample results for the $B_{1}^{+}$ maps from the 7T knee coil on multiple slices inside the phantom for 6 arbitrary shims. The comparisons were made on slices close to the center of each element type: (a) Loops and (b) Monopoles. Each column presents results for one shim. Left and right groups are the results for the $B_{1}^{+}$‐driven and the S‐matrix‐driven optimization methods, respectively. The absolute error results were included for a visual comparison.

Figure 8 shows a similar comparison for the 10.5T head coil for arbitrary RF shims. In Figure 8, shim #1 is CP, shim #2 is ZP, and shims #3 and #4 are random magnitude and phase shims. Additional comparisons showing $B_{1}^{+}$ fields on all slices compared against measurements using both methods are provided in the Supporting Information Figures S4–S7. Also, the optimized variables by applying the $B_{1}^{+}$‐driven and the S‐matrix‐driven optimization methods: attenuation $A_{n}$, phase shift $\Theta_{n}$, inductance $L_{n}$, and capacitance $C_{n}$ are given in Figure S8 for the 7T and 10.5T arrays. Generally speaking, the values for the lumped elements values obtained from the two methods were similar, which indicates that both methods are valid for improving the simulation modeling accuracy. However, the attenuators and phase shifters results are totally different since the objectives for each method are also different.

**FIGURE 8:**
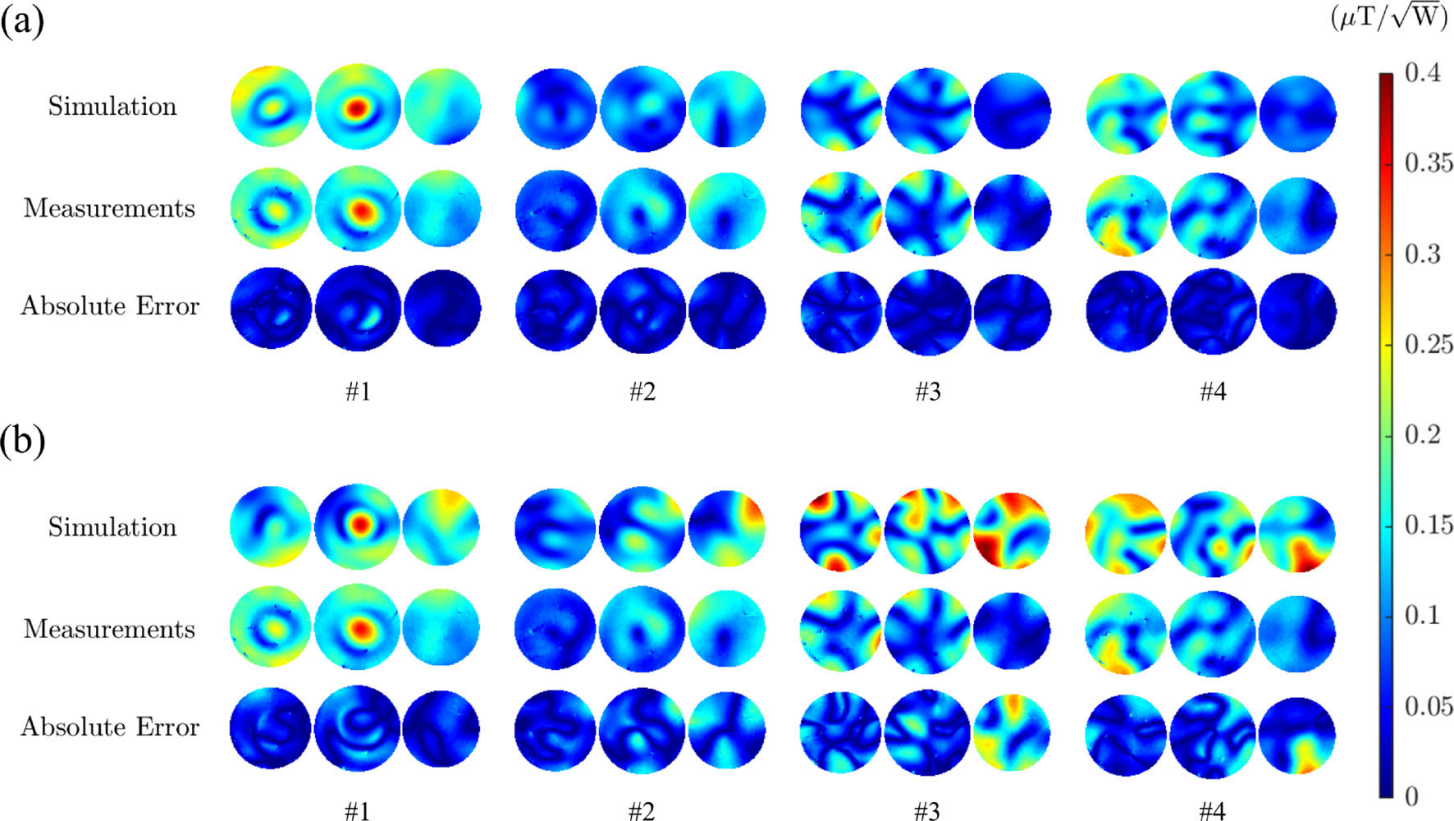
Sample results for the $B_{1}^{+}$ maps from the 10.5T head coil on multiple slices inside the phantom for 4 arbitrary shims. The comparisons were made on 2 slices close to the center of each row in addition to another slice at the center between the two rows (total of 3 slices). (a) and (b) results are for the $B_{1}^{+}$‐driven and the S‐matrix‐driven methods, respectively. The absolute error results were included for a visual comparison.

The statistical distributions of the obtained $B_{1}^{+}$‐fields NRSME against measurements using 50,000 random magnitude and phase shims are given in Figure 9. These results clearly demonstrate the advantage of the $B_{1}^{+}$‐driven method over the S‐matrix‐driven method in terms of accuracy. For the 7T knee coil, the average NRMSE were 30.8% and 48.0%, for the $B_{1}^{+}$‐driven and S‐matrix‐driven methods, respectively. For the 10.5T head coil, the average NRMSE were 50.9% and 91.1%, for the $B_{1}^{+}$‐driven and the S‐matrix‐driven methods, respectively. Figure 10 shows a validation for the obtained 10g average SAR using both the $B_{1}^{+}$‐driven and S‐matrix‐driven methods compared to measurements using three random RF shims. The corresponding NRMSE were 0.19, 0.17, and 0.27 using the $B_{1}^{+}$‐driven method. And 0.18, 0.38, and 0.58 using the S‐matrix‐driven method.

**FIGURE 9:**
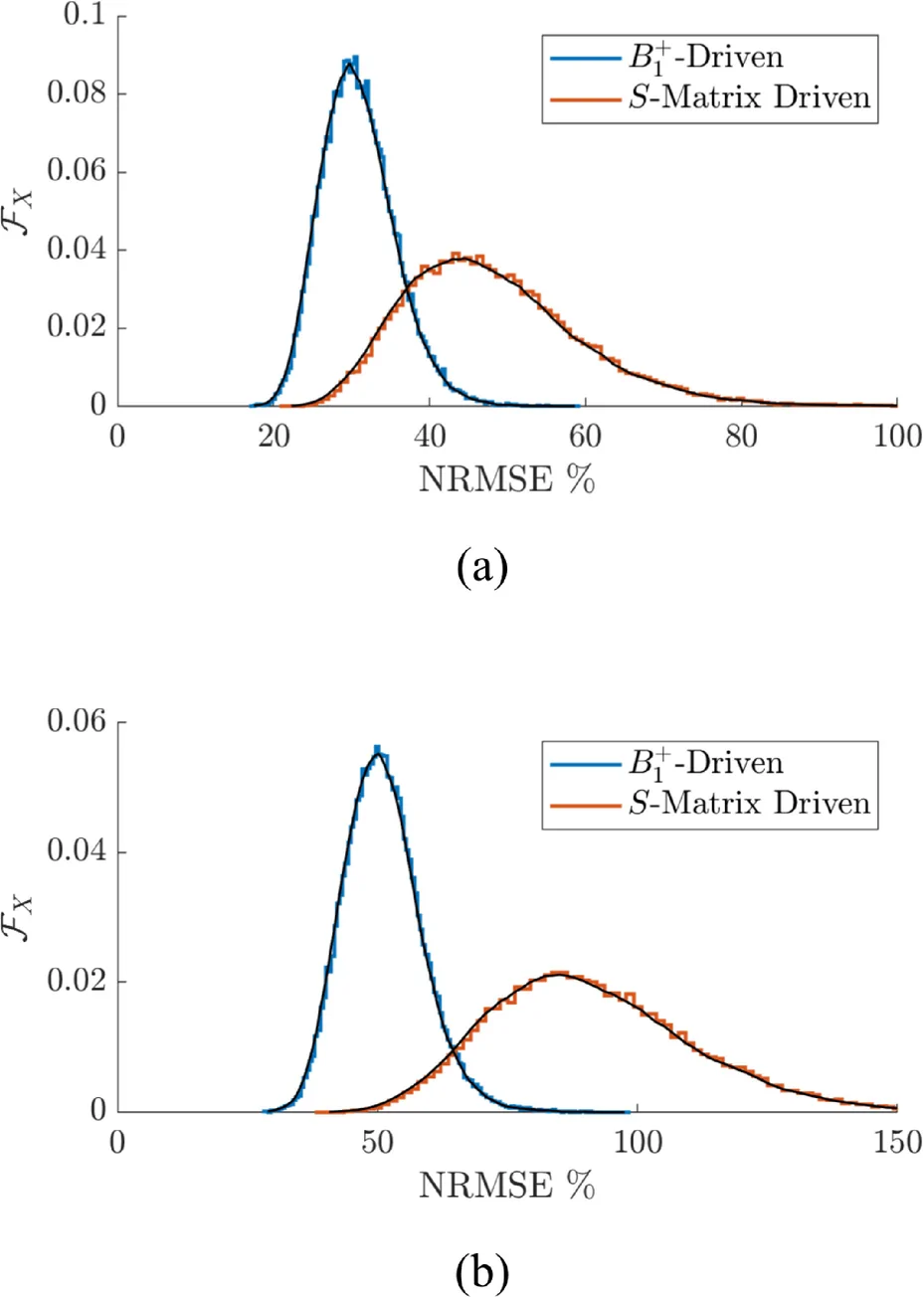
The statistical distributions for the $B_{1}^{+}$ results NRMSE obtained by the $B_{1}^{+}$‐driven and the S‐matrix‐driven methods for (a) the 7T knee coil and (b) the 10.5T head coil. A total of 50,000 random magnitude and phases shims were applied to each optimized model. For the knee coil, the mean values were 30.8% and 48.0%, while the 99.9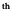 percentiles were 48.9% and 98.8% for the $B_{1}^{+}$‐driven and the S‐matrix‐driven methods, respectively. For the head coil, the mean values were 50.9% and 91.1%, while the 99.9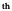 percentiles were 83.0% and 170.2% for the $B_{1}^{+}$‐driven and the S‐matrix‐driven methods, respectively. The RMSE were normalized to the average value of the measured $B_{1}^{+}$ maps. The black line is a moving average interpolation for each dataset.

**FIGURE 10:**
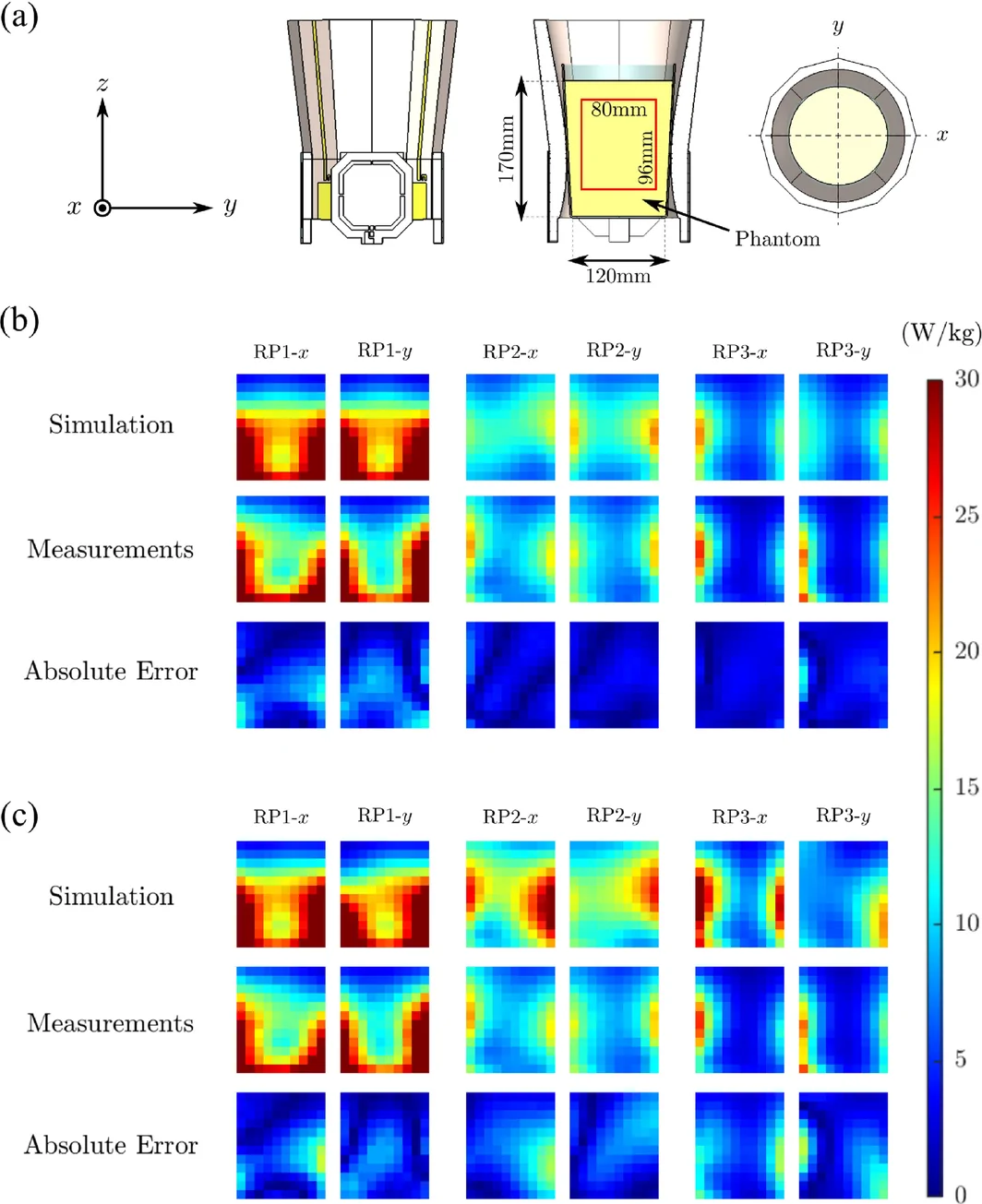
A validation for the 10g average SAR inside a blunted cone shaped phantom on two orthogonal vertical planes normal to x‐axis or y‐axis. (a) An illustration for the setup and the phantom. (b) and (c) are the 10g average SAR results from three random phase (RP) RF shims illustrated on each plane using the $B_{1}^{+}$‐driven and S‐matrix driven methods, respectively.

## 4 Discussion and Conclusions

The comparison between the two methods shows a clear advantage for the proposed $B_{1}^{+}$‐driven RF co‐simulation optimization approach illustrating the utility of the presented formulations. Accurate EM simulations producing EM fields as close as possible to the physically implemented RF coil, can reduce the need for large safety margins, providing more flexible RF management without sacrificing patient safety. When comparing the results from each method against the measurements, the $B_{1}^{+}$‐driven method clearly outperformed the S‐matrix‐driven method in capturing the experimentally measured $B_{1}^{+}$ maps and 10g average SAR both with reduced NRMSE. Further investigations could focus on optimization of any additional cost functions for assessing modeling error such as those proposed in previously published work [30]. The proposed method can accommodate possible effects from factors such as MR bore and patient's bed, radiation losses, and additional losses in the scanner which are not easy to describe with S‐matrix measurement on the bench. Although, the S‐matrix implicitly includes these effects, it is usually impractical or not feasible to obtain these measurements inside the MR scanner environment.

One limitation of this $B_{1}^{+}$‐driven approach is that it only evaluates the $B_{1}^{+}$ errors for modeling purposes. In UHF MRI, peak 10g local SAR is the most widely used metric to assess RF safety. Although it is possible to measure SAR in a phantom using either Magnetic Resonance Thermometry (MRT) or fiber‐optic temperature probes, it is generally easier and faster to quantify modeling errors through $B_{1}^{+}$‐fields comparisons. Furthermore, previous works have demonstrated that modeling error can be estimated using both $B_{1}^{+}$ and SAR [24, 30, 31, 32]. Therefore, in this work, we chose to focus on a $B_{1}^{+}$‐driven approach. In principle, the proposed RF co‐simulation approach could also be implemented to minimize the error between measured and modeled SAR. However, SAR‐driven optimization would require a significantly large number of SAR patterns. Such patterns may be captured using a setup capable of directly measuring the Q‐matrices. This potentially valuable approach requires further investigation. Although, not investigated here, the proposed $B_{1}^{+}$‐driven approach can be deployed for designing RF coils by setting the optimization targets to obtain a desired $B_{1}^{+}$‐field distribution. This can result in an expedited design process while allowing the RF coil designer to explore various design choices and optimization techniques taking into account the RF co‐simulation network effects. Additionally, the number of RF ports in the EM simulation can increase if one decides to embed each lumped element as a tunable variable. Although this approach is very common in MRI RF coil simulation, we recommend moving all the matching networks to the co‐simulation in order to reduce the EM simulation overhead. This way, the number of RF ports in the EM simulation (internal ports) does not increase.

The statistical results from Figure 9 clearly describe the advantages of the proposed methods within this work. An important observation is that both the mean value and the variance were different for the error histograms calculated using the $B_{1}^{+}$‐driven and the S‐matrix‐driven methods. Assuming a normal distribution for the NRMSE $X∼𝒩(\mu ,\sigma^{2})$, when increasing the error by a factor of α>1, then X becomes $X∼𝒩(\alpha \mu ,\alpha^{2}\sigma^{2})$. Thus, it is expected to obtain a narrower statistical distribution for the NRMSE when reducing the error in $B_{1}^{+}$. Moreover, the application of many random shims implicitly validated the physical relevance of the obtained model since the model was only optimized for a few selected RF shims. Of course, one could include a larger number of RF shims in the optimization, but this can lead to a very slow calculation. Also, it is possible to introduce Q‐values for the lumped elements, which is more realistic, and tune these values as independent variables for the optimization. Moreover, combining measurements on multiple phantoms with different EM properties can provide a more rigorous way to improve the accuracy of the models. Additionally, one can try different cost functions implementations and different optimizers for faster and better convergence, which can be a topic for another investigation. On the other hand, the 10g average SAR results in Figure 10 suggest that the proposed method has clearly improved the EM modeling error by directly evaluating the variation in the SAR maps in contrast to the $B_{1}^{+}$ field maps. However, this also suggests further validations and investigations in the direction of defining the SAR patterns as the ultimate goal for the optimization, which introduces new technical challenges.

Another important feature of the proposed method is the speed of computation. The evaluation of the combined fields is only performed on the desired slices with a flexible choice for the resolution. For example, the time it takes to compute the fields from a single RF shim for the 7T knee coil using the formulation described here took less than 100 msec. On the other hand, a frequently used professional commercial software (CST Studio) took several minutes to compute the combined fields for the same coil. Although one needs to take into account that these results depend on many factors, including the number of internal RF ports, the size of the simulation domain, and the requested fields monitors, the difference in computing time is substantial. The numbers above are reported to give a realistic estimate for a practical scenario since they are based on the simulation model presented in this work. Moreover, the proposed method provides total flexibility in choosing the desired slices and resolution when performing the $B_{1}^{+}$‐driven optimization, which also contributes to expedited numerical calculations.

In conclusion, the presented RF co‐simulation optimization framework produces a better agreement between measured and simulated $\left|B_{1}^{+}\right|$ patterns. This improvement allows for the use of smaller safety factors. Future studies including additional SAR and temperature measurements can help further investigate the accuracy of the proposed approach.

## Funding

This work was supported by the National Institutes of Health (NIH) under grants P41 EB027061, R01 NS115180, and R01 EB033365.

## Conflicts of Interest

The authors declare no conflicts of interest.

## Supporting information




**Figure S1.** The optimized S‐matrix phases from the S‐matrix‐driven method compared to the measured S‐matrix for the 7T knee coil and the 10.5T head coil on the top and bottom rows, respectively. First column is the measured S‐matrix data. The second column is an optimized co‐simulated S‐matrix. The last column is the error between the measured and co‐simulated S‐matrix.
**Figure S2.** The resulting channel‐wise $B_{1}^{+}$ maps on slices inside the phantom compared to the measurements for the 10.5T head coil obtained using the $B_{1}^{+}$‐driven optimization method. The comparisons were made on slices close to the center of each row.
**Figure S3.** The resulting channel‐wise $B_{1}^{+}$ maps on slices inside the phantom compared to the measurements for the 10.5T head coil obtained using the S‐matrix‐driven optimization method. The comparisons were made on slices close to the center of each row.
**Figure S4.** Sample results for the 7T knee coil on all slices using (a) CP and (b) ZP shims. The left column is the $B_{1}^{+}$‐driven method and the right column is the S‐matrix‐driven method.
**Figure S5.** Sample results for the 7T knee coil on all slices using two random shims in (a) and (b). Left column is the $B_{1}^{+}$‐driven method and right column is the S‐matrix‐driven method.
**Figure S6.** Sample results for the 10.5T head coil on all slices using (a) CP and (b) ZP shims. Left column is the $B_{1}^{+}$‐driven method and right column is the S‐matrix‐driven method.
**Figure S7.** Sample results for the 10.5T head coil on all slices using two random shims in (a) and (b). Left column is the $B_{1}^{+}$‐driven method and right column is the S‐matrix‐driven method.
**Figure S8.** The final values for the optimized variables on the co‐simulation using the $B_{1}^{+}$‐driven and the S‐matrix‐driven methods for the 7T knee and 10.5T head coils. For the knee coil: (a) The 32 capacitors values. (b) The 4 inductors values. (c) The ideal attenuators values. (d) The ideal phase shifters values. For the head coil: (e) The ideal attenuators values. (f) The ideal phase shifters values. (g) The 80 capacitors values.

## Data Availability

The authors have nothing to report.

## Appendix A The Star (⋆) Product

The star product S‐matrix concatenation can be used for cascading multiple lumped elements to create practical matching networks [33]. Assuming two 2‐port networks a and b to be cascaded, the overall S‐matrix is given by the star product as

(A1)
$$S=S^{a}⋆S^{b},$$

where

(A2)
$$S_{1,1}=S_{1,1}^{a}+S_{1,2}^{a}S_{1,1}^{b}{\left[1-S_{2,2}^{a}S_{1,1}^{b}\right]}^{-1}S_{2,1}^{a},$$

(A3)
$$S_{1,2}=S_{1,2}^{a}{\left[1-S_{1,1}^{b}S_{2,2}^{a}\right]}^{-1}S_{1,2}^{b},$$

(A4)
$$S_{2,1}=S_{2,1}^{b}{\left[1-S_{2,2}^{a}S_{1,1}^{b}\right]}^{-1}S_{2,1}^{a},$$

and

(A5)
$$S_{2,2}=S_{2,2}^{b}+S_{2,1}^{b}S_{2,2}^{a}{\left[1-S_{1,1}^{b}S_{2,2}^{a}\right]}^{-1}S_{1,2}^{b}.$$

For creating arbitrary networks, one can cascade series and shunt elements using the definitions [34]

(A6)
$$S_{\text{series}}=\frac{1}{Z+2Z_{0}}\left[\begin{array}{ll} Z & 2Z_{0}\\ 2Z_{0} & Z \end{array}\right],$$

and

(A7)
$$S_{\text{shunt}}=\frac{1}{Y+2Y_{0}}\left[\begin{array}{ll} -\;Y & 2Y_{0}\\ 2Y_{0} & -Y \end{array}\right],$$

where $Y_{0}=1/Z_{0}$. On the other hand, for a lumped element termination impedance $Z_{n}$ corresponding to a reflection coefficient $\Gamma_{L}$

(A8)
$$S^{\left(n\right)}=\frac{Z_{n}-Z_{0n}}{Z_{n}+Z_{0n}}\left[\begin{array}{ll} 0 & 0\\ 0 & 1 \end{array}\right]=\left[S_{1,1}+\frac{S_{1,2}S_{2,1}\Gamma_{L}}{1-S_{2,2}\Gamma_{L}}\right]\left[\begin{array}{ll} 0 & 0\\ 0 & 1 \end{array}\right].$$

An ideal attenuator $A_{n}$ (in dB) cascaded with a phase shifter $\Theta_{n}$ (in radian) can be combined using $S^{\left(n\right)}=\left[\begin{array}{ll} 0 & 1\\ 1 & 0 \end{array}\right]10^{A_{n}/20}e^{j\Theta_{n}}$.
