# Supplementary material for: A Fast B1+‐Driven RF Co‐Simulation Optimization Method for RF Coil Arrays
Source: Magn Reson Med. 2026 Sep 29;97(1):441–53. doi: 10.1002/mrm.70603 (PMC13746108; doi:10.1002/mrm.70603)
Supplement: Supplementary file 1 [file MRM-97-441-s001.pdf]

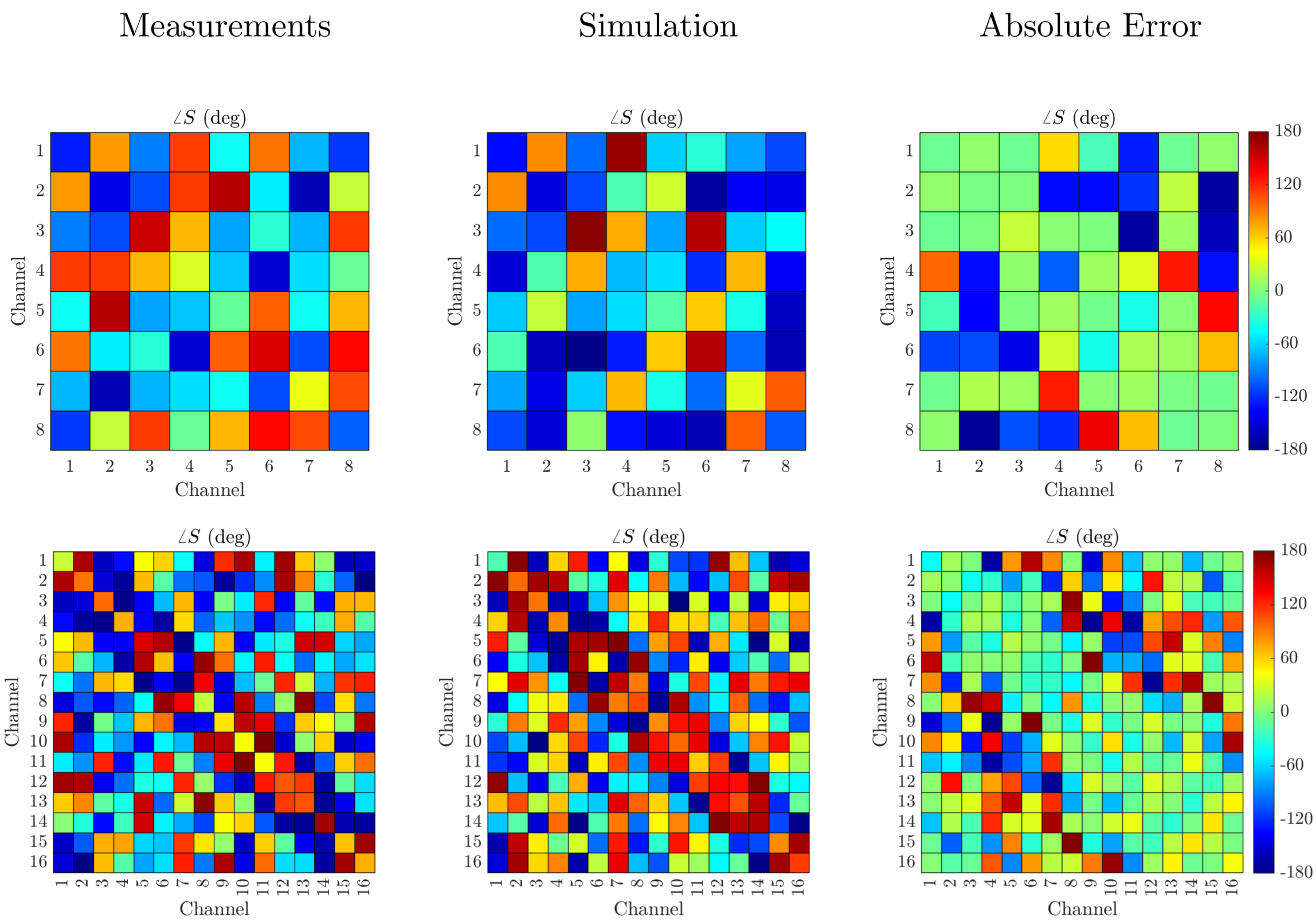

Figure S1: The optimized S-matrix phases from the S-matrix driven method compared to the measured S-matrix for the 7T knee coil and the 10.5T head coil on the top and bottom rows, respectively. First column is the measured S-matrix data. Second column is optimized co-simulated S-matrix. The last column is the error between the measured and co-simulated S-matrix.

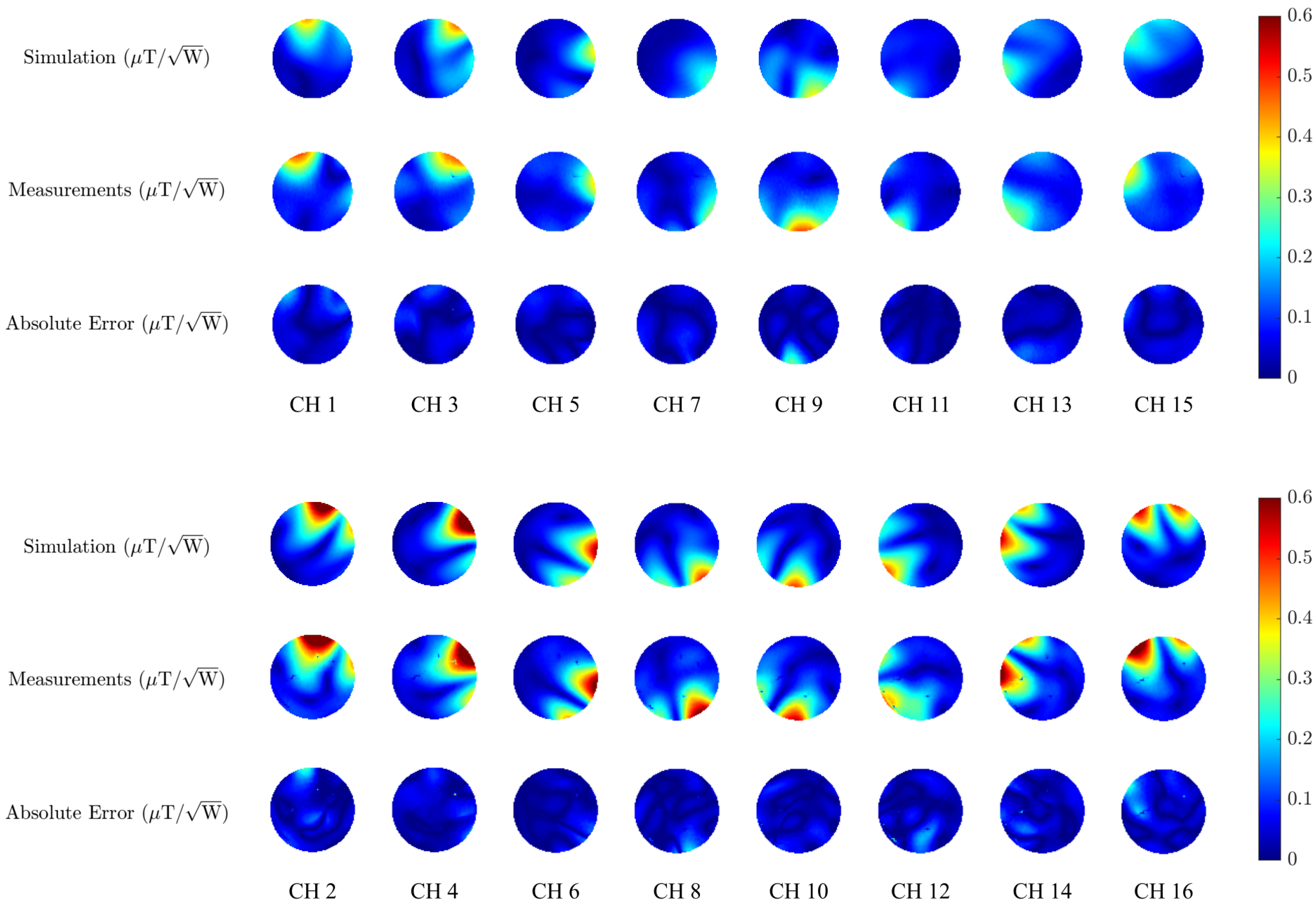

Figure S2: The resulting channel-wise B_1^+ maps on slices inside the phantom compared to the measurements for the 10.5T head coil obtained using the B_1^+-driven optimization method. The comparisons were made on slices close to the center of each row.

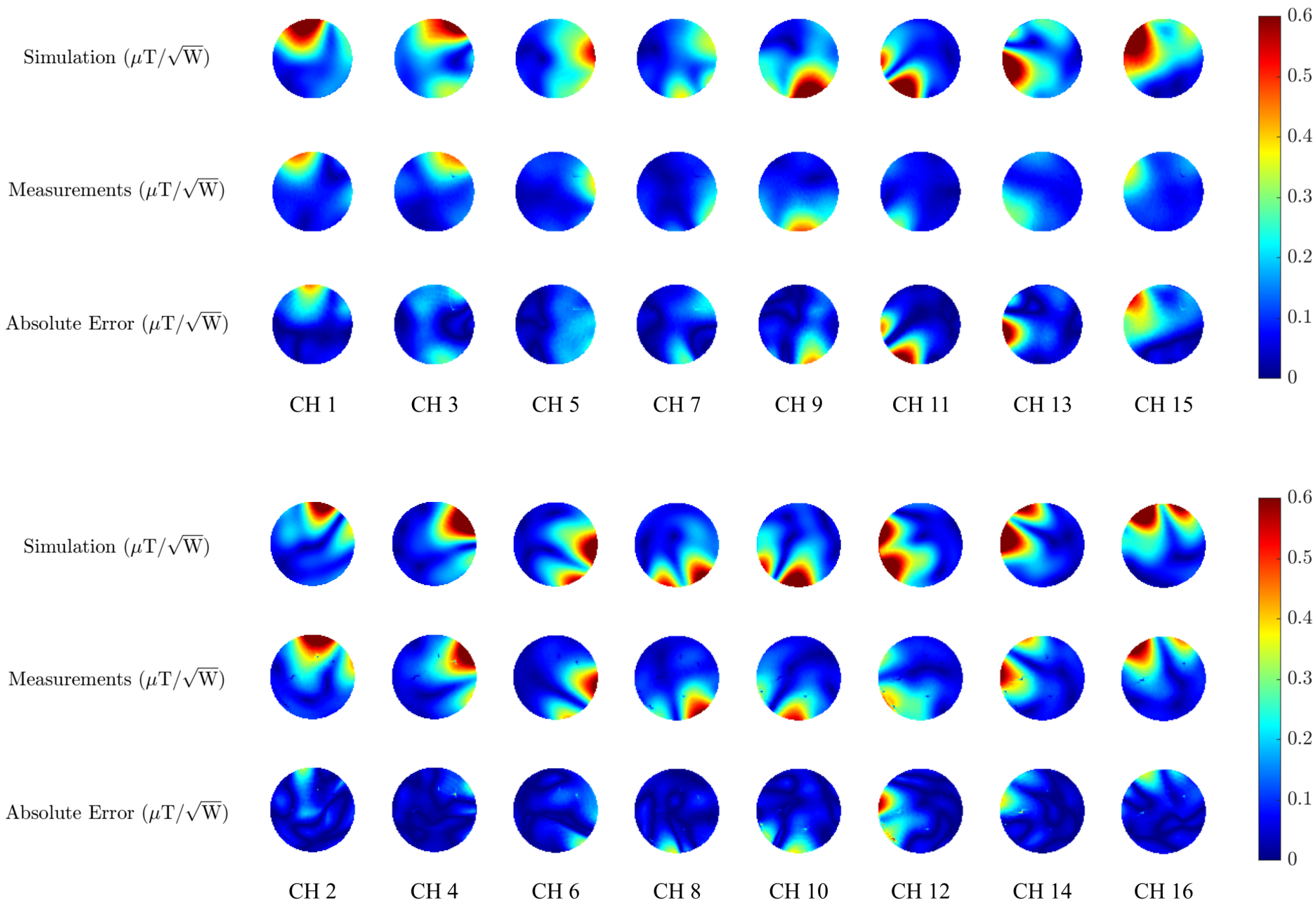

Figure S3: The resulting channel-wise B_1^+ maps on slices inside the phantom compared to the measurements for the 10.5T head coil obtained using the S-matrix driven optimization method. The comparisons were made on slices close to the center of each row.

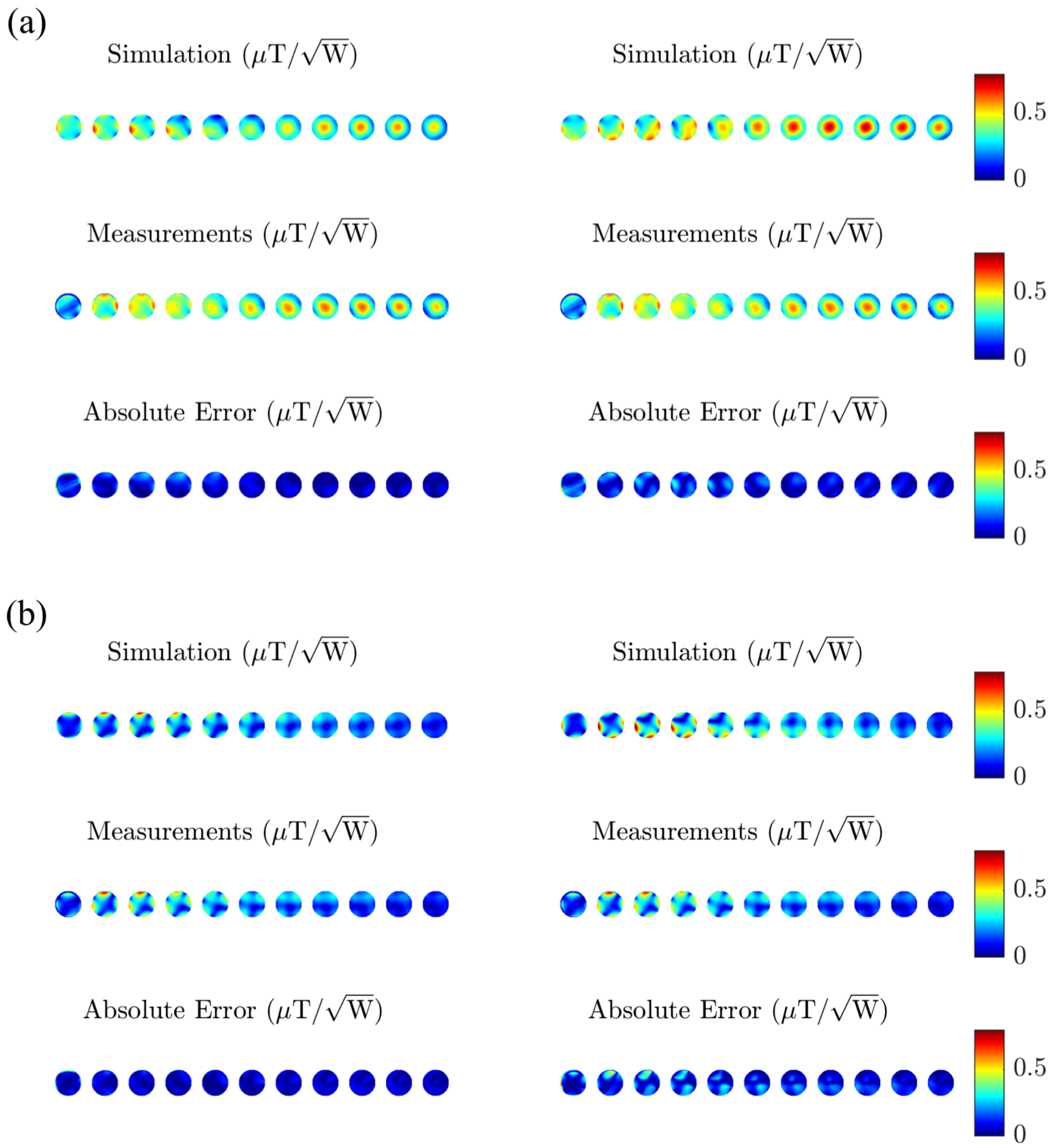

Figure S4: Sample results for the 7T knee coil on all slices using (a) CP and (b) ZP shims. Left column is the B_1^+-driven method and right column is the S-matrix driven method.

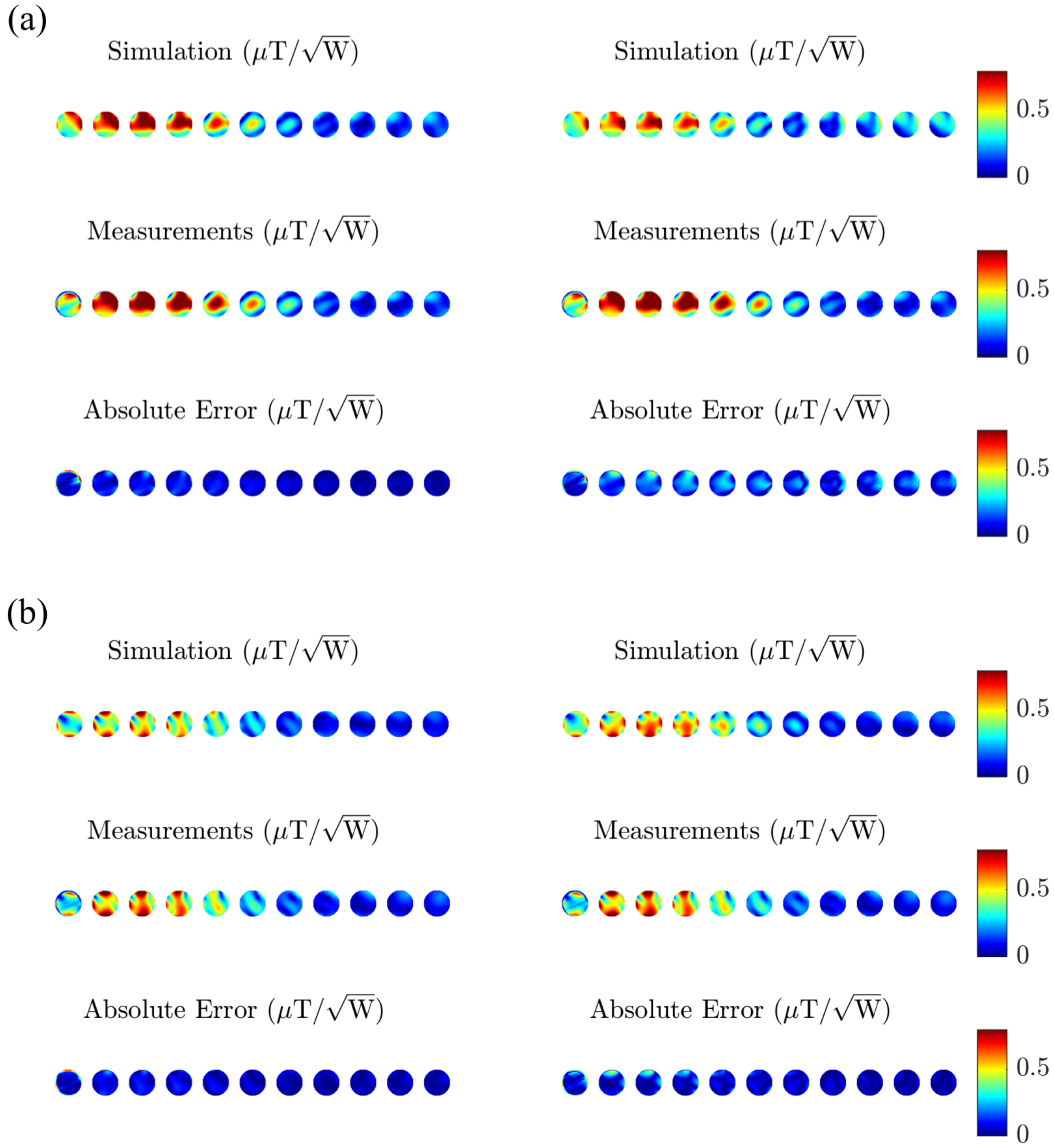

Figure S5: Sample results for the 7T knee coil on all slices using two random shims in (a) and (b). Left column is the B_1^+-driven method and right column is the S-matrix driven method.

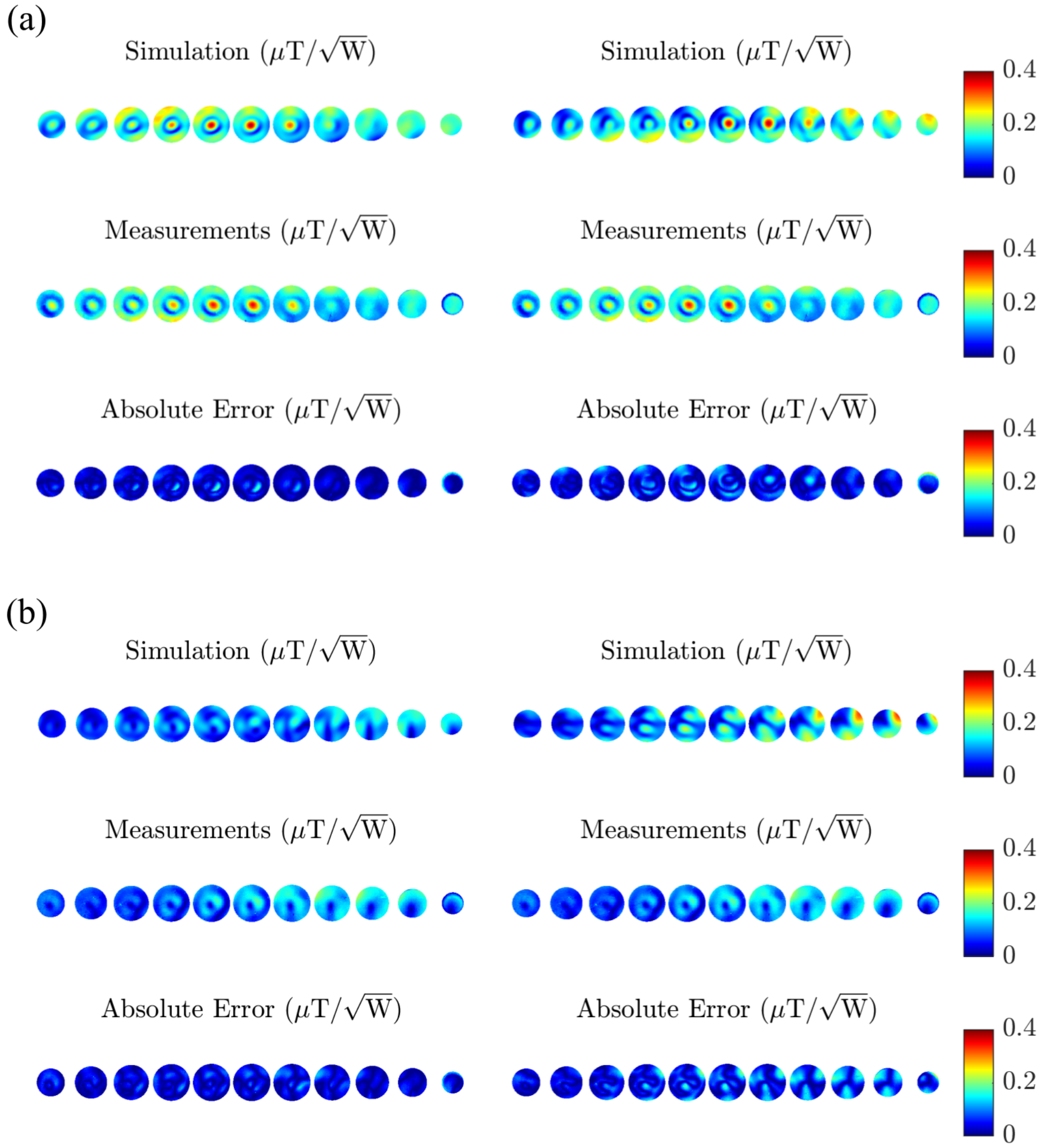

Figure S6: Sample results for the 10.5T head coil on all slices using (a) CP and (b) ZP shims. Left column is the B_1^+-driven method and right column is the S-matrix driven method.

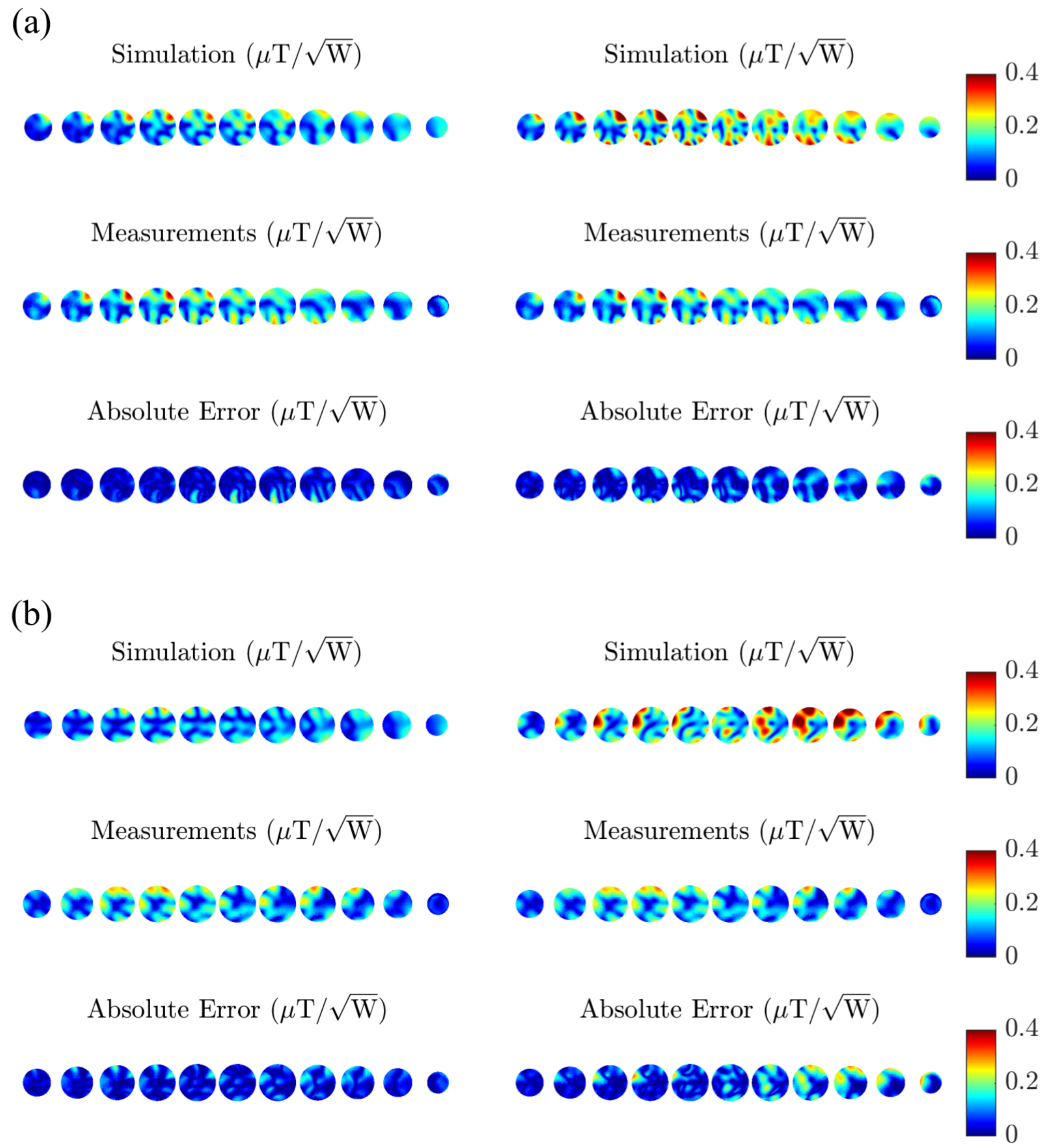

Figure S7: Sample results for the 10.5T head coil on all slices using two random shims in (a) and (b). Left column is the B_1^+-driven method and right column is the S-matrix driven method.

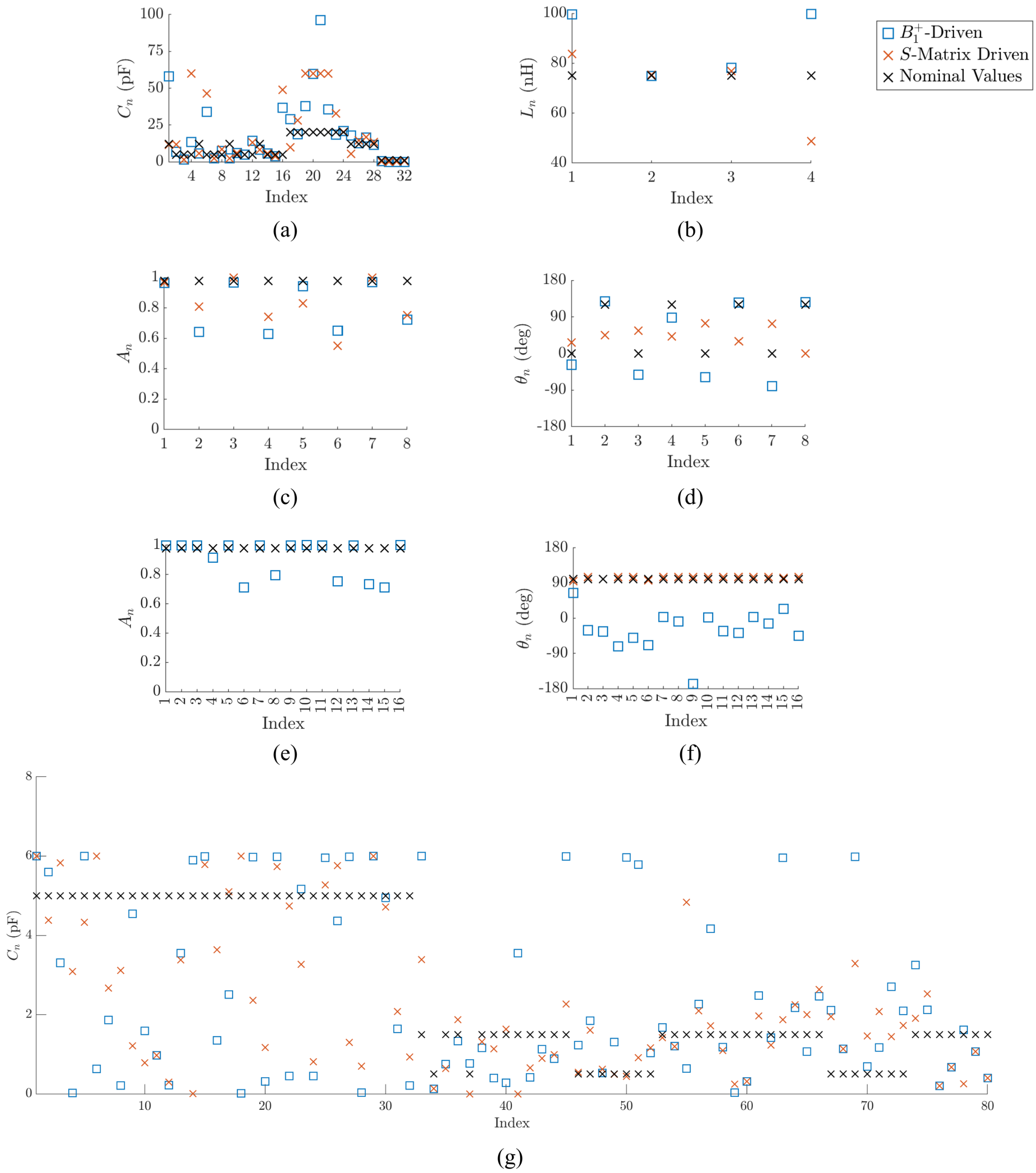

Figure S8: The final values for the optimized variables on the co-simulation using the B_1^+-driven and the S-matrix driven methods for the 7T knee and 10.5T head coils. For the knee coil: (a) The 32 capacitors values. (b) The 4 inductors values. (c) The ideal attenuators values. (d) The ideal phase shifters values. For the head coil: (e) The ideal attenuators values. (f) The ideal phase shifters values. (g) The 80 capacitors values.
